# Under-recognition of heart failure in patients with atrial fibrillation and the impact of gender: a UK population-based cohort study

**DOI:** 10.1186/s12916-021-02048-8

**Published:** 2021-08-10

**Authors:** Rosita Zakeri, Ann D. Morgan, Varun Sundaram, Chloe Bloom, John G. F. Cleland, Jennifer K. Quint

**Affiliations:** 1 Department of Population Science and Gene Health, National Heart and Lung Institute, Imperial College London, London, UK; 2 School of Cardiovascular Medicine & Sciences, King’s College London British Heart Foundation Centre for Research Excellence, 125 Coldharbour Lane, London, SE5 9NU UK; 3 Department of Cardiovascular Medicine, Harrington Heart and Vascular Institute, University Hospitals Cleveland Medical Center, Case Western Reserve University, Cleveland, OH USA; 4 Robertson Centre for Biostatistics and Clinical Trials, University of Glasgow, Glasgow, UK

**Keywords:** Atrial fibrillation, Loop diuretics, Heart failure, Epidemiology

## Abstract

**Background:**

Patients with atrial fibrillation (AF) complicated by heart failure (HF) have a poor prognosis. We investigated whether long term loop-diuretic therapy in patients with AF and no known diagnosis of HF, as a potential surrogate marker of undiagnosed HF, is also associated with worse outcomes.

**Methods:**

Adults with incident AF were identified from UK primary and secondary care records between 2004 and 2016. Repeat prescriptions for loop diuretics, without a diagnosis of HF or documented non-cardiac indication, were classified as ‘isolated’ loop diuretic use.

**Results:**

Amongst 124,256 people with incident AF (median 76 years, 47% women), 22,001 (17.7%) had a diagnosis of HF, and 22,325 (18.0%) had isolated loop diuretic use. During 2.9 (LQ-UQ 1–6) years’ follow-up, 12,182 patients were diagnosed with HF (incidence rate 3.2 [95% CI 3.1–3.3]/100 person-years). Of these, 3999 (32.8%) had prior isolated loop diuretic use, including 31% of patients diagnosed with HF following an emergency hospitalisation. The median time from AF to HF diagnosis was 3.6 (1.2–7.7) years in men versus 5.1 (1.8–9.9) years in women (p = 0.0001). In adjusted models, patients with isolated loop diuretic use had higher mortality (HR 1.42 [95% CI 1.37–1.47], p < 0.0005) and risk of HF hospitalisation (HR 1.60 [95% CI 1.42–1.80], p < 0.0005) than patients with no HF or loop diuretic use, and comparably poor survival to patients with diagnosed HF.

**Conclusions:**

Loop diuretics are commonly prescribed to patients with AF and may indicate increased cardiovascular risk. Targeted evaluation of these patients may allow earlier HF diagnosis, timely intervention, and better outcomes, particularly amongst women with AF, in whom HF appears to be under-recognised and diagnosed later than in men.

**Supplementary Information:**

The online version contains supplementary material available at 10.1186/s12916-021-02048-8.

## Background

Atrial fibrillation (AF) is a common cardiac arrhythmia associated with substantial morbidity. With contemporary anticoagulation strategies, heart failure (HF) has now replaced stroke as the most common non-fatal adverse outcome amongst patients with AF [1, 2]. Hence, better prevention and management of HF have become key priorities of AF care [3]. Early identification of HF would provide an opportunity to commence guideline-recommended HF therapy, which may reduce the risk of decompensation, hospitalisation, and improve survival [4]. However, the diagnosis of HF can be challenging in patients with AF, particularly in the primary care setting [5, 6]. Typical symptoms and elevated plasma natriuretic peptides may be attributed to AF rather than HF [7–9], and the accuracy of cardiovascular imaging may be diminished [10], increasing the likelihood of a delayed or missed HF diagnosis.

Fluid retention and congestion are common clinical manifestations of HF, not directly related to AF, which may be treated with loop-diuretic agents. Many HF patients require regular doses of loop-diuretics in order to relieve HF-related symptoms and maintain a euvolaemic state [4]. In addition, loop-diuretics may be prescribed for a number of non-cardiac indications, and their use potentially associated with electrolyte disturbances or neuro-endocrine activation that can precipitate cardiorenal syndromes or progression of subclinical to overt HF.

We hypothesised that a subset of patients with AF and no known HF, who require long-term loop-diuretic therapy, may have unrecognised early-stage HF and poor outcomes, parallel or worse than AF patients with known HF. To investigate this hypothesis, we determined the prevalence and incidence of HF and loop-diuretic use in a large, population-based cohort of patients with newly diagnosed AF. Patients who received repeat prescriptions for loop-diuretic agents, with no recorded HF diagnosis or common non-cardiac indication for loop-diuretic therapy, were defined as having ‘isolated’ loop-diuretic use. Patient characteristics and outcomes, including all-cause mortality, emergency HF hospitalisation, and stroke, were compared between patients with AF with and without recognised HF.

## Methods

### Data source

This population-based cohort study was performed using prospectively collected data from the primary care UK Clinical Practice Research Datalink (CPRD) linked to secondary care records from Hospital Episodes Statistics (HES) and national death registration data (ONS). The CPRD contains anonymised primary care electronic health records for more than 11.3 million individuals and has been demonstrated to be broadly representative of the UK population with respect to age, gender, and ethnicity [11]. The CPRD performs a quality check for each primary care practice to ensure that records are accurate and reliable; only data labelled by CPRD as ‘up-to-standard’ [11] were included in this study. Ethical approval for observational research using the CPRD has been granted by a Health Research Authority Research Ethics Committee (East Midlands-Derby, REC reference number 05/MRE04/87). Our research protocol complies with the Declaration of Helsinki and was approved by the Independent Scientific Advisory Committee for MHRA Database Research.

### Study population and AF ascertainment

We extracted data for men and women (age ≥ 18 years), registered for at least 1 year in CPRD, and approved for HES and ONS linkage, between January 1, 2004, and December 31, 2016. Incident AF or flutter was identified by the earliest reported diagnostic code in the CPRD (read code) or at hospital discharge (International Classification of Diseases, Tenth Revision; ICD-10 code), excluding patients with congenital heart disease (Additional file 1: Supplemental methods p. 2–5).

The prevalence of loop-diuretic use and/or diagnosed HF at the time of AF diagnosis was recorded. Patients without diagnosed HF or isolated loop-diuretic use formed the referent population for baseline comparisons. Secondly, to study the incidence of HF, only patients without a record of HF by the time of AF diagnosis were included, and the primary exposure was assigned as the earliest recording of loop-diuretic prescription or HF, whichever came first (Additional file 1: Supplemental methods p. 2–3).

### Exposures and outcomes

At baseline (i.e., time of AF diagnosis), four exposure groups were defined as: (i) no HF or loop-diuretic use, (ii) isolated loop-diuretic use, (iii) diagnosed HF without loop-diuretic use, and (iv) diagnosed HF with loop-diuretic use. Heart failure was defined by the presence of a HF-related diagnostic code in either primary (CPRD) or secondary care (HES, code lists provided in Additional file 1: Supplemental methods p. 6–12) and subclassified as HF occurring prior to (> 3 months before) or concurrent with (± 3 months) incident AF. An overlap of ± 3 months was chosen to minimise misclassification bias by allowing time for information to flow between hospitals and primary care. Long-term loop-diuretic use was defined as a minimum of 3 consecutive prescriptions, within 100 days, with the first prescription taken as the start date for loop-diuretic use. Patients without a diagnosis of HF who had advanced kidney disease (stage 5 and renal replacement therapy), nephrotic syndrome, or chronic liver disease, as identified by the relevant diagnostic codes in primary or secondary care, were considered to have a primary non-cardiac indication for loop-diuretic use in the main analysis. Hypertension alone, without HF, was not considered to be an indication for loop-diuretic use, since guidelines for antihypertensive stipulate therapy with thiazide rather than loop-diuretics [12]. Other individuals with loop-diuretic prescriptions, including all patients with a documented HF diagnosis, were assumed to have a probable cardiac indication. ‘Isolated’ loop-diuretic use was defined as long-term loop-diuretic use, for a presumed cardiac indication, with no recorded diagnosis of HF.

The index date for the start of follow-up was the date of AF diagnosis. Patients without diagnosed HF or isolated loop-diuretic use at the time of AF diagnosis formed the referent population for ascertainment of incident HF and isolated loop-diuretic use as outcome measures (i.e., occurring > 3 months after AF diagnosis; Additional file 1: Supplemental methods p. 3). Other outcomes examined for the overall cohort included all-cause mortality (ONS), hospitalisation due to HF (HES Admitted Patient Care dataset; ICD-10 code for HF recorded in the first or second position), and ischaemic stroke or systemic thromboembolism (recorded in primary or secondary care). In the main analysis, we excluded patients with less than 100 days of follow-up (due to death or censoring) as these individuals could not fulfil our definition of long-term diuretic use. Patients not experiencing the respective outcome were censored at their last date of primary or secondary care contact, de-registration with the primary care practice or study end date (December 31, 2016), as relevant.

### Statistical analysis

Baseline groups were defined according to the presence or absence of exposure (either HF diagnosis or isolated loop-diuretic use) at AF diagnosis (Table 1). Group data are presented as frequency (%), mean ± SD, or median (lower quartile-upper quartile). Between-group comparisons were made using the Pearson χ^2^ test for categorical variables and 1-way ANOVA or Kruskal-Wallis test for continuous variables.

**Table 1:** Patient characteristics at AF diagnosis

Characteristic	Total population with incident AF, n = 124,256	Heart failure at AF diagnosis			
		No, n = 102,255 (82.3%)		Yes, n = 22,001 (17.7%)	
		Treated with loop diuretic?			
		No	Yes^**a**^	No	Yes^**a**^
Number of individuals (% total)		79,930 (64.3)	22,325 (18.0)	6,082 (4.9)	15,919 (12.8)
Age (y)	76 (68, 83)	74 (65, 81)	80 (73, 86)	76 (67, 83)	79 (71, 85)
Women, n (%)	58,222 (46.9)	35,111 (43.9)	13,250 (59.4)	2407 (39.6)	7454 (46.8)
Systolic blood pressure (mmHg)	133 (120, 145)	135 (122, 145)	133 (120, 144)	130 (120, 142)	130 (116, 140)
Missing data, n (%)	1,459 (1.2)	1,175 (1.5)	146 (0.7)	74 (1.2)	64 (0.4)
Diastolic blood pressure (mmHg)	79 (70, 84)	80 (70, 85)	78 (70, 82)	78 (70, 84)	75 (67, 80)
Missing data, n (%)	1459 (1.2)	1175 (1.5)	146 (0.7)	74 (1.2)	64 (0.4)
BMI (kg/m^2^)	28.1 ± 6.2	27.8 ± 5.9	29.1 ± 6.9	27.7 ± 5.9	28.6 ± 6.6
BMI category^b^, n (%)					
Underweight	2647 (2.1)	1643 (2.1)	527 (2.7)	133 (2.2)	344 (2.2)
Normal	26,909 (21.7)	17,598 (22.0)	4306 (19.3)	1433 (23.6)	3572 (22.4)
Overweight	33,401 (26.9)	22,155 (27.7)	5441 (24.4)	1659 (27.2)	4146 (26.0)
Obese	30,765 (24.8)	17,871 (22.4)	6952 (31.1)	1386 (22.8)	4556 (28.6)
Missing data	30,534 (24.6)	20,663 (25.9)	5126 (23.0)	1471 (24.1)	3301 (20.7)
Smoking, n (%)					
Current smoker	35,106 (28.3)	22,008 (27.5)	6192 (27.7)	1864 (30.7)	5042 (31.7)
Ex-smoker	36,115 (29.1)	22,487 (28.1)	6888 (30.9)	1736 (28.5)	5004 (31.4)
Total	71,221 (57.3)	44,495 (55.7)	13,080 (58.6)	3,600 (59.2)	10,046 (63.1)
Comorbidities, n (%)					
Cardiovascular					
Hypertension	81,137 (65.3)	48,601 (60.8)	16,924 (75.8)	3933 (64.7)	11,679 (73.4)
Ischaemic heart disease	37,286 (30.0)	17,765 (22.2)	8316 (37.3)	2661 (43.8)	8544 (53.7)
Prior myocardial infarction	15,208 (12.2)	6236 (7.8)	3206 (14.4)	1379 (22.7)	4387 (27.6)
Stroke/TIA	17,744 (14.3)	11,035 (13.8)	3520 (15.8)	859 (14.1)	2330 (14.6)
Valvular heart disease	14,412 (11.6)	5958 (7.5)	3264 (14.6)	1328 (21.8)	3862 (24.3)
PPM/ICD	3008 (2.4)	1397 (1.8)	525 (2.4)	234 (3.9)	852 (5.4)
Peripheral artery disease	10,367 (8.3)	5387 (6.7)	2218 (9.9)	652 (10.7)	2110 (13.3)
Dyslipidaemia	32,477 (26.1)	19,242 (24.1)	6272 (28.1)	1798 (29.6)	5165 (32.5)
Respiratory					
Asthma	20,198 (16.3)	11,386 (14.2)	4544 (20.4)	990 (16.3)	3278 (20.6)
COPD	14,943 (12.0)	7072 (8.9)	3796 (17.0)	801 (13.2)	3274 (20.6)
Interstitial lung disease	2776 (2.2)	1399 (1.8)	639 (2.9)	177 (2.9)	561 (3.5)
Obstructive sleep apnoea	1464 (1.2)	804 (1.0)	327 (1.5)	57 (0.9)	276 (1.7)
Renal					
End-stage kidney disease	2286 (1.8)	1456 (1.8)	-	160 (2.6)	670 (4.2)
Nephrotic syndrome	259 (0.2)	193 (0.2)	-	6 (0.1)	60 (0.4)
Other					
Diabetes	20,684 (16.7)	10,839 (13.6)	4943 (22.1)	945 (15.5)	3957 (24.9)
Chronic liver disease	1325 (1.1)	994 (1.2)	-	89 (1.5)	242 (1.5)
Malignancy (any)	21,076 (17.0)	13,149 (16.5)	4174 (18.7)	1021 (16.8)	2732 (17.2)
Malignancy (top 4 causes)^c^	5438 (4.4)	3470 (4.3)	1012 (4.5)	294 (4.8)	662 (4.2)
Depression	18,565 (14.9)	11,413 (14.3)	3691 (16.5)	879 (14.5)	2582 (16.2)
CHA_2_DS_2_-VASc score	3.3 ± 1.7	2.8 ± 1.6	3.7 ± 1.4	4.1 ± 1.7	4.6 ± 1.5
Medication, n (%)					
ACEI	64,855 (52.2)	33,333 (41.7)	14,414 (64.6)	3982 (65.5)	13,126 (82.4)
ARB	7435 (6.0)	4394 (5.5)	1668 (7.5)	339 (5.6)	1034 (6.5)
ACEI or ARB	72,290 (58.2)	37,727 (47.2)	16,082 (72.0)	4321 (71.1)	14,160 (89.0)
ARNI	< 5 (0.0)	-	-	-	< 5 (0.0)
MRA	9277 (7.5)	1273 (1.6)	2241 (10.0)	633 (10.4)	5130 (32.2)
Antiarrhythmic drug^d^	11,195 (9.0)	5898 (7.4)	2174 (9.7)	758 (12.5)	2365 (14.9)
Beta-blocker	81,591 (65.7)	50,136 (62.7)	15,290 (68.5)	4293 (70.6)	11,872 (74.6)
Digoxin	30,309 (24.4)	13,619 (17.0)	7758 (34.8)	1802 (29.6)	7130 (44.8)
Dihydropyridine CCB	50,994 (41.0)	29,683 (37.1)	11,496 (51.5)	2889 (41.2)	6926 (46.2)
Non-dihydropyridine CCB	16,588 (13.4)	8733 (10.9)	4151 (18.6)	752 (12.4)	2952 (18.5)
Anticoagulant therapy	58,348 (47.0)	34,750 (43.5)	11,523 (51.6)	3055 (50.2)	9020 (56.7)
Antiplatelet therapy	86,018 (69.2)	52,364 (65.5)	17,048 (76.4)	4163 (68.5)	12,443 (78.2)
Loop diuretic (non-cardiac indication)	1125 (0.9)	1125 (1.4)	-	-	-
Statin	65,336 (52.6)	38,803 (48.9)	13,103 (58.7)	3336 (54.9)	10,094 (63.4)

Data presented as mean (SD), median (LQ, UQ), or frequency (%) as appropriate

*BMI* body mass index, *COPD* chronic obstructive pulmonary disease, *ICD* implantable cardioverter defibrillator, *PPM* permanent pacemaker, *TIA* transient ischaemic attack

^a^Refers to loop-diuretic therapy for a presumed cardiac indication. Patients on loop-diuretic therapy with end-stage renal failure, chronic liver disease, or nephrotic syndrome (without a diagnosis of heart failure) were considered to have a non-cardiac indication and therefore included in the ‘no loop-diuretic therapy’ category for this analysis. Patients with heart failure and loop-diuretic use were assumed to have a cardiac indication

^b^BMI was categorised as underweight (< 18.5 kg/m^2^), normal (18.5–24.9 kg/m^2^), overweight (25–29.9 kg/m^2^), and obese (≥ 30 kg/m^2^)

^c^Aggregated number of cases of the four most common causes of cancer (lung, breast, bowel, and prostate) as reported by Cancer Research UK in 2015 (Cancer Research UK, Cancer incidence statistics, https://www.cancerresearchuk.org/health-professional/cancer-statistics/incidence; accessed Sep. 26, 2019).

^d^Excluding non-dihydropyridine calcium channel blockers

Outcomes were examined between 1st January 2004 and 31st December 2016. In patients without diagnosed HF or isolated loop-diuretic use at AF diagnosis, the crude incidence of HF or new isolated loop-diuretic use was calculated from the number of new cases divided by the sum of all individual person-years in the cohort. Standardised incidence rates were computed based on the age and gender distribution of the UK population in CPRD in 2011 [13]. The Aalen-Johansen estimator was used to compute overall and gender-stratified cumulative incidence curves for diagnosed HF and isolated loop-diuretic use, and death before HF as competing risks.

Survival was estimated with the Kaplan-Meier method and compared between baseline exposure groups using the log-rank test. Cox regression models were used to assess the association between baseline group and all-cause mortality, unplanned (emergency) hospitalisation due to HF, and ischaemic stroke in sequential models: (i) model 1, unadjusted; (ii) model 2, adjusted for age, gender, systolic blood pressure, body mass index (BMI), smoking status, and comorbidities at AF diagnosis: COPD, diabetes, hypertension, previous MI and previous stroke or TIA; and (iii) model 3, adjusted for model 2 variables and baseline medication use at AF diagnosis (ACEI or ARB, beta-blocker, MRA, digoxin, anticoagulant, antiplatelet, antiarrhythmic drugs, non-dihydropyridine CCB, and statin use). The Fine and Gray method was used to compute death as a competing risk for non-fatal outcomes. Patients without a diagnosis of HF and not taking loop-diuretics were used as the referent population. Effect modification by gender was tested by including an interaction term between exposure group and gender and using the likelihood ratio test to assess its significance. Where an interaction was found, gender-stratified Cox regressions were performed to examine the association of baseline group with outcomes in women and men separately. Missing data were handled by available case analyses. The proportional hazard assumption was examined graphically and using formal tests (as described by Grambsch et al. [14]). No major deviations from this assumption were observed.

### Sensitivity analyses

First, all patients without a fourth prescription for loop-diuretic therapy within 5 years were re-classified as ‘no loop-diuretic use’ thereby excluding patients with a single (recovered) episode of HF and confirming the validity of our findings for patients with chronic HF (versus acute and chronic HF combined). Second, all patients with a recorded diagnosis of valvular heart disease or a history of valvular heart surgery were excluded, to select a cohort of patients with non-valvular AF. Third, we removed the (100-day) censor period after AF diagnosis to confirm whether a confounding effect would be observed when including patients with an early death (or loss to follow-up) who would be unable to be coded as diuretic positive. Fourth, we extended the censor period to 1 year, to investigate reverse causality (i.e., late-stage HF causing AF). Fifth, we excluded patients in whom loop-diuretics were initiated within 12 months of death, in order to minimise reverse causation due to alternative non-HF life-limiting indications (although this would also exclude late presenting or advanced HF). Sixth, due to a relatively high rate of missing BMI data, we explored the association between isolated loop-diuretic use and outcomes restricted to patients without a BMI recording. Seventh, in some patients with advanced CKD, loop-diuretics may be used in preference to thiazide diuretics for the treatment of hypertension. Therefore, we excluded patients with stage 3 and 4 chronic kidney disease from the isolated loop-diuretic group (in addition to stage 5 and renal replacement therapy), in order to examine potential confounding due to a non-HF-related loop-diuretic indication in this patient group. Finally, to examine the risks associated with exposure (i.e., isolated loop-diuretic use or HF diagnosis) occurring at any time during follow-up, we substituted baseline exposure groups with a time-dependent definition of exposures, i.e., patients were categorised as exposed at the time of first loop-diuretic use or HF diagnosis at any time during follow-up, and unexposed before that.

Study findings are reported in accordance with The Strengthening the Reporting of Observational Studies in Epidemiology (STROBE) Statement [15]. Notably, we report our results in relation to gender rather than sex, as per the Sex and Gender Equality in Research (SAGER) guidelines [16], because UK primary care records are based on self-identified and reported gender rather than biological sex. Analyses were performed using STATA MP (version 15.0; StataCorp LLC, TX).

## Results

### Study population

**Fig. 1:**
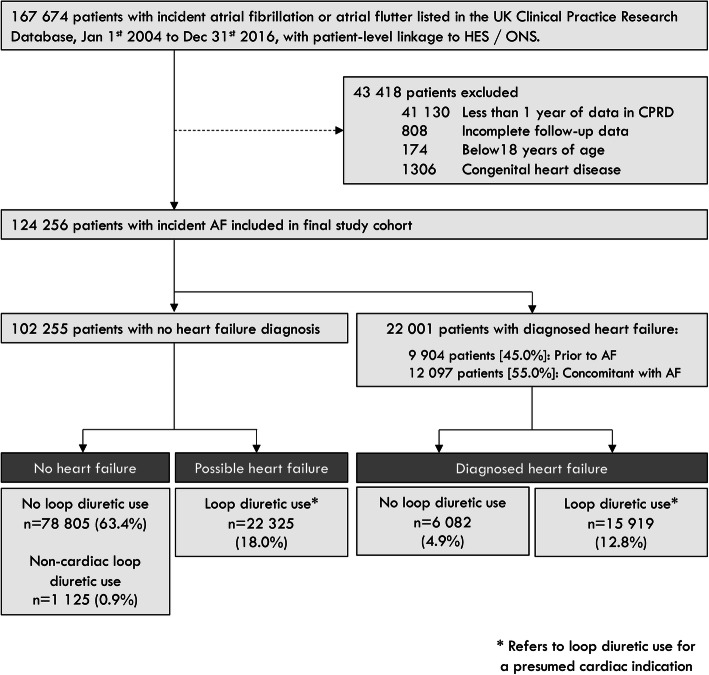
Participant flow diagram. Isolated loop-diuretic use refers to loop-diuretic use for a presumed cardiac indication and in the absence of a diagnosis of heart failure. Percentages represent the proportion of the final study cohort (n = 124,256)

We identified 124,256 adults with incident AF between 2004 and 2016 (Fig. 1). The median (LQ-UQ) age at AF diagnosis was 76 (68–83) years and 47% were women. Patient characteristics are shown in Table 1.

At AF diagnosis, 22,001 (17.7%) patients had a prior or concomitant diagnosis of HF (Fig. 1, Table 1). A further 22,325 (18.0%) patients had ‘isolated’ loop-diuretic use. Patients with isolated loop-diuretic use were, on average, older, more likely to be women, with a BMI ≥ 30 kg/m^2^ (obese range) and hypertension, as compared to patients with no HF or diagnosed HF (Table 1). Apart from a lower prevalence of coronary and peripheral artery disease, isolated loop-diuretic use was associated with a similar comorbidity burden to patients with diagnosed HF. The median number of loop-diuretic prescriptions before the onset of AF was 6 (LQ-UQ 2-28), reflecting approximately 6 months’ prevalent use. Rates of anticoagulation were similar between patients with isolated loop-diuretic use and diagnosed HF; however, ACEI or ARBs, beta-blockers, and mineralocorticoid receptor antagonists (MRAs) were prescribed less often for patients with isolated loop-diuretic use.

**Fig. 2:**
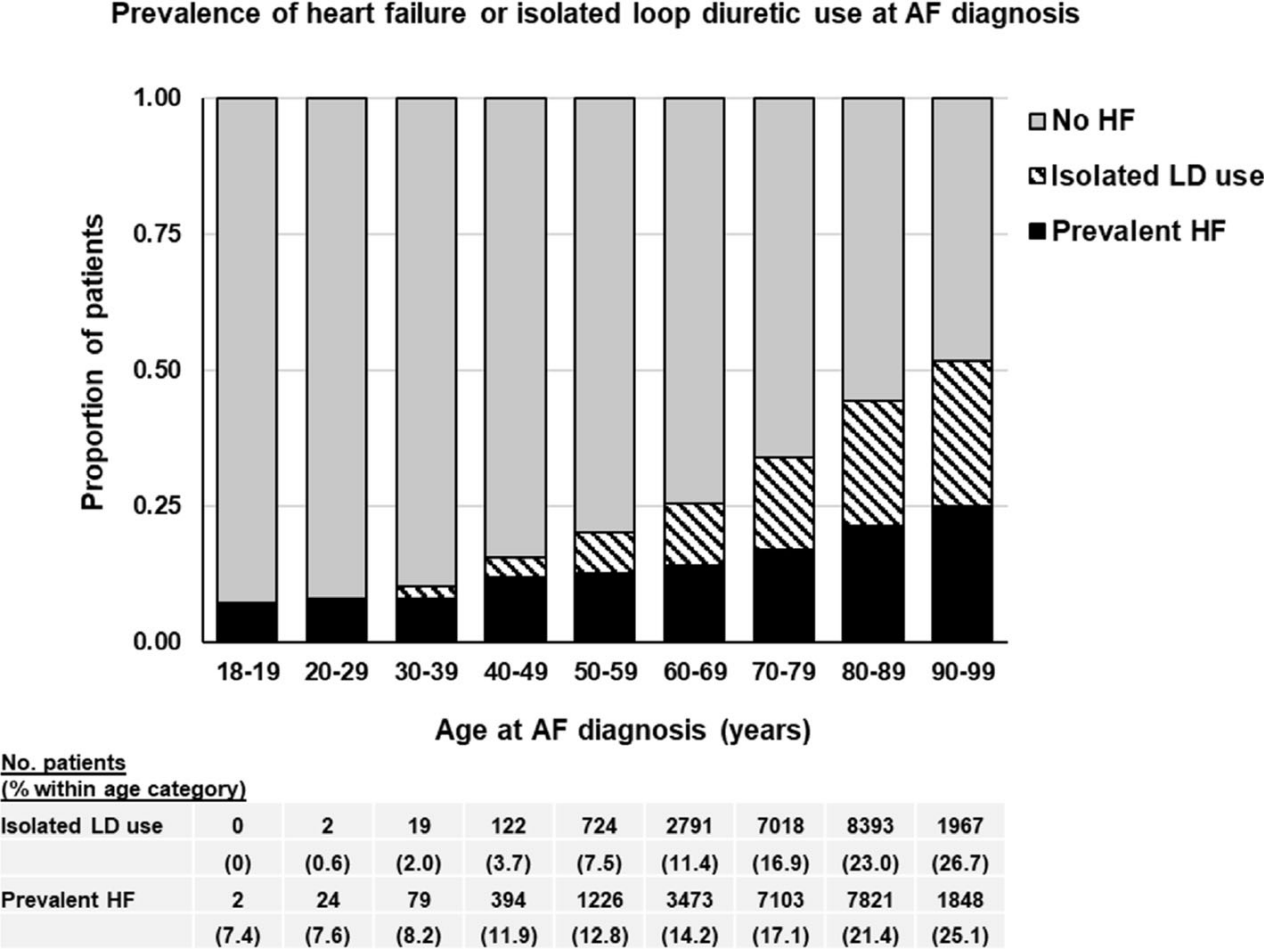
Prevalence of diagnosed heart failure and isolated loop-diuretic use by age at AF diagnosis. Includes heart failure recorded any time before, and up to 3 months after, the first record of AF

The prevalence of isolated loop-diuretic use was highest in older age groups, mirroring diagnosed HF (Fig. 2), and did not change significantly over the study period (Additional file 2: Supplemental Figure 1). Isolated loop-diuretic use was more common in women than men (Additional file 2: Supplemental Table 3), particularly women over 50 years of age (Additional file 2: Supplemental Figure 2).

### Incidence of heart failure

**Fig. 3:**
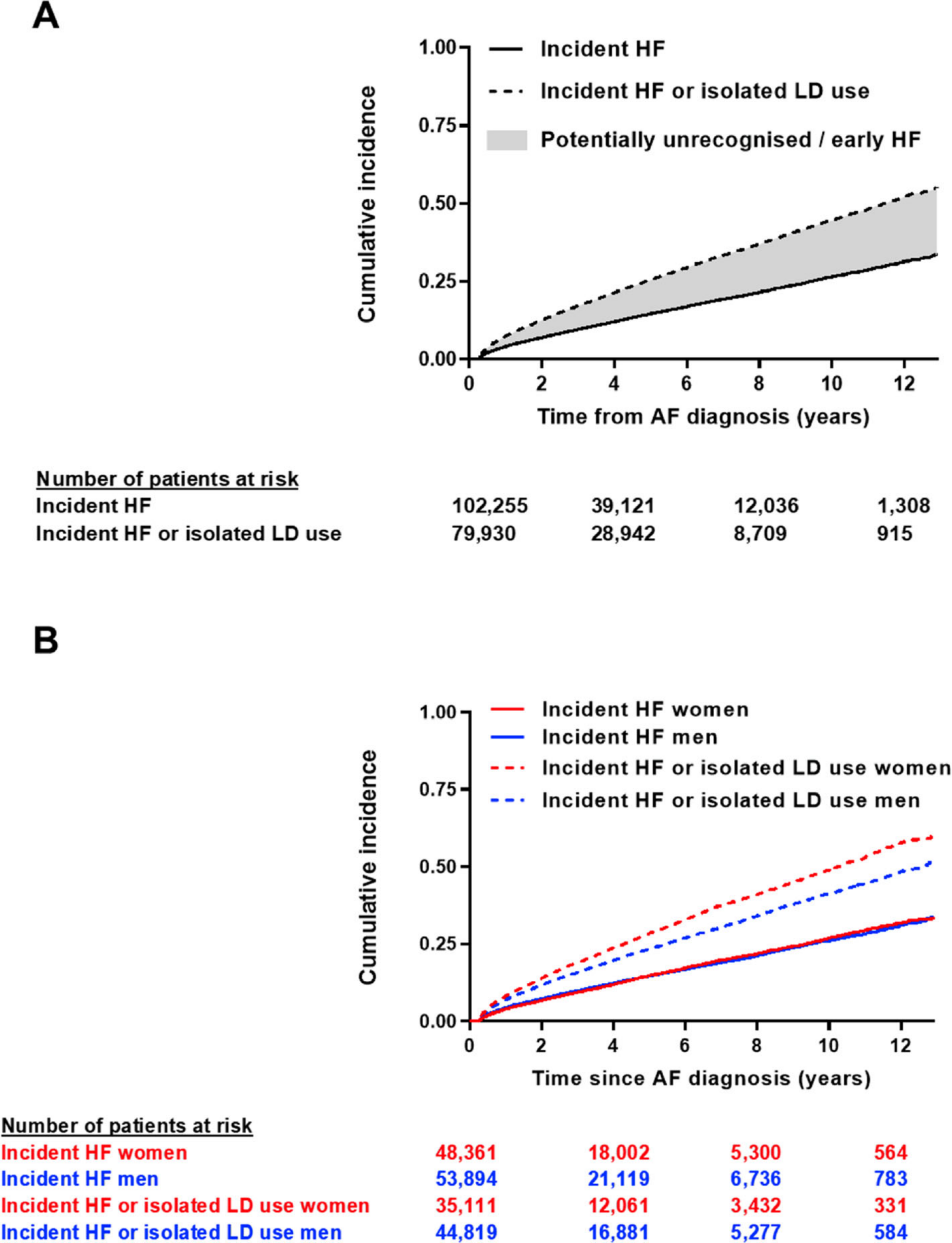
Cumulative incidence of diagnosed heart failure and isolated loop-diuretic use. **A** Total population. **B** Stratified by gender. Diagnosed heart failure represented by a solid line, diagnosed heart failure and isolated loop-diuretic use combined represented by a dashed line

Amongst 102,255 patients with AF without prevalent HF, 12,182 new cases of HF were diagnosed over a median follow-up of 2.9 years (LQ-UQ 1–6 years), giving a crude HF incidence of 3.2 (95% CI 3.1–3.3) events per 100 person-years (Additional file 2: Supplemental Table 1). Almost one third (n = 3999; 32.8%) of patients with a new diagnosis of HF had prior ‘isolated’ loop-diuretic use, including 1228/3964 (31%) patients in whom HF was diagnosed following an unplanned (emergency) hospitalisation. An additional 9349 patients were initiated on long-term loop-diuretics sometime after AF onset, with no HF diagnosis ever recorded. When combined with diagnosed HF, the overall incidence of isolated loop-diuretic use or diagnosed HF was 6.1 (95% CI 6.0–6.2) events per 100 person-years (Fig. 3A, Additional file 2: Supplemental Table 1).

On gender-stratified analyses, the crude incidence of diagnosed HF was similar for men and women (Fig. 3B), although women had a higher competing risk of death (Additional file 2: Supplemental figure 3). A greater proportion of women received their HF diagnosis following an unplanned hospitalisation (34% women versus 31% men, p = 0.001) and, amongst patients with antecedent diuretic use, the median time interval between first loop-diuretic prescription, and recorded HF diagnosis was 5.1 (1.8–9.9) years in women versus 3.6 (1.2–7.7) years in men (p = 0.0001). When isolated loop-diuretic use was combined with diagnosed HF, the overall incidence was greater for women than for men (Fig. 3B, Additional file 2: Supplemental Table 1) and the median time interval between AF diagnosis and earliest potential HF presentation (either HF diagnosis or first loop-diuretic prescription) was similar between genders (women 2.1 [0.8–4.4] years versus men 2.1 [0.8–4.5] years, p = 0.061).

### Death, heart failure hospitalisation, and stroke

**Fig. 4:**
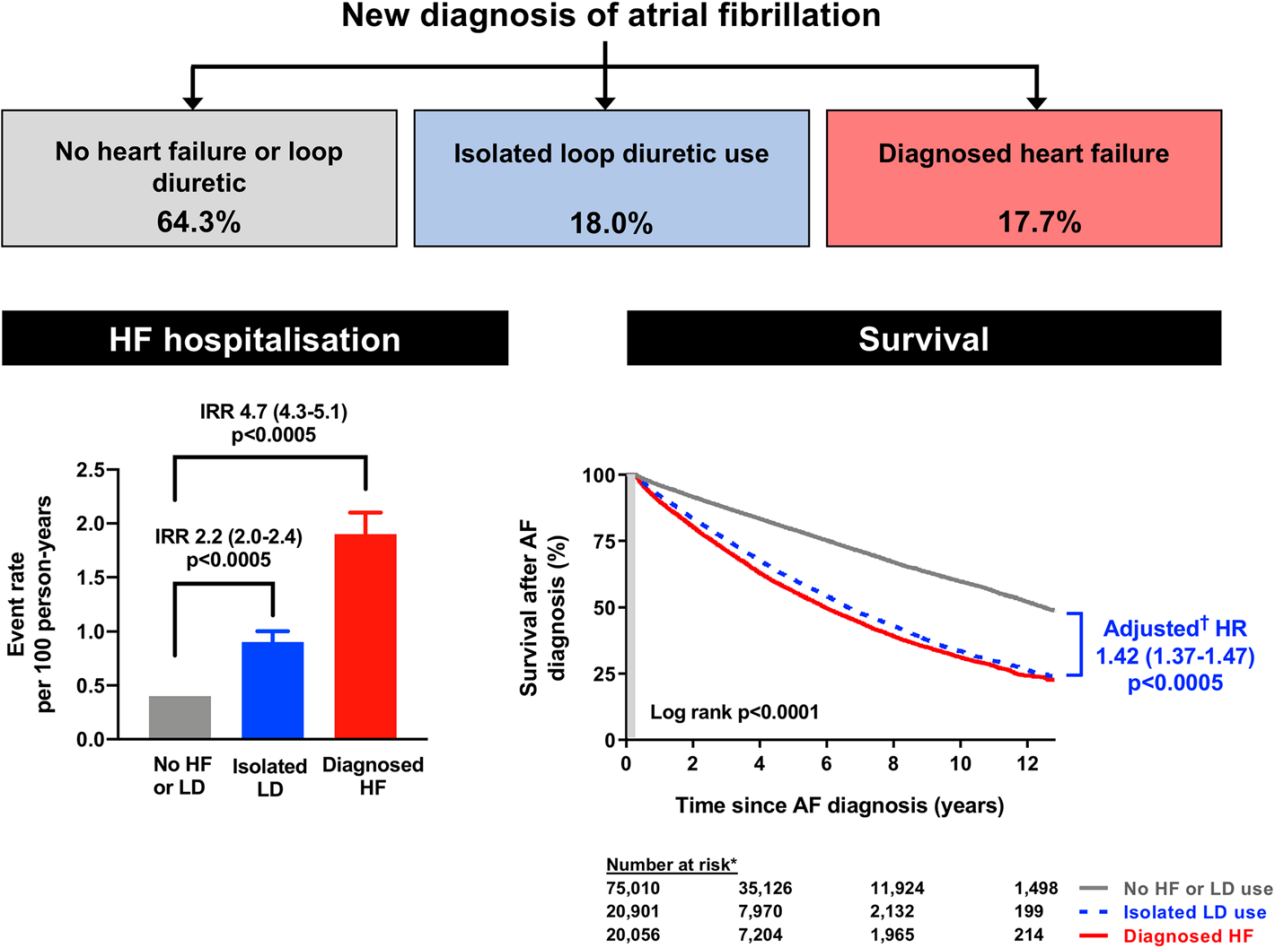
Left panel: Rates of unplanned (emergency) hospitalisation for heart failure per 100 person-years of follow-up. Right panel: Survival after AF diagnosis, according to baseline heart failure status. AF, atrial fibrillation; HF, heart failure; IRR, incidence rate ratio; LD, loop-diuretic (refers to loop-diuretic use for a presumed cardiac indication). Asterisk (*) indicates the number of patients at risk for each baseline group, after 100-day censor period. † indicates data adjusted for age, gender, systolic blood pressure, BMI, smoking status, and comorbidities (COPD, diabetes, hypertension, previous MI, previous stroke/TIA) and medication use at AF diagnosis (ACEI/ARB, beta-blocker, MRA, digoxin, anticoagulant, antiplatelet, antiarrhythmic drug, non-dihydropyridine CCB, and statin use)

Overall, 32,258 (26.0%) patients died over a median follow-up of 3.1 (LQ-UQ 1.3–5.9) years (Additional file 2: Supplemental Table 2). Survival was poorest amongst patients with a baseline diagnosis of HF and loop-diuretic use (Fig. 4, Table 2). Mortality was also higher amongst patients with isolated loop-diuretic use versus patients with no HF diagnosis or loop-diuretic use (Fig. 4), which persisted after adjustment for age, gender, baseline comorbidities, and medication use (Table 2). Isolated loop-diuretic use was associated with more frequent unplanned HF hospitalisation than no loop-diuretic use (Fig. 4, Table 2). In addition, isolated loop-diuretic use as well as diagnosed HF was associated with a reduced risk of stroke after multivariable adjustment, accounting for the competing risk of death (Table 2).

**Table 2:** Risk of adverse outcomes after AF diagnosis, by baseline heart failure status

Outcome (n = 124,256)	Model 1^a^	Model 2^b^	Model 3^c^
	HR (95% CI)	HR (95% CI)	HR (95% CI)
All-cause mortality			
No HF or LD use	Referent	Referent	Referent
Isolated LD use	2.10 (2.04–2.15)***	1.45 (1.41–1.51)***	1.42 (1.37–1.47)***
Diagnosed HF	1.54 (1.47–1.63)***	1.36 (1.28–1.45)***	1.33 (1.26–1.42)***
Diagnosed HF + LD use	2.79 (2.71–2.87)***	1.94 (1.87–2.00)***	1.78 (1.71–1.85)***
Unplanned HF hospitalisation^Δ^			
No HF or LD use	Referent	Referent	Referent
Isolated LD use	1.90 (1.73–2.09)***	1.63 (1.45–1.83)***	1.60 (1.42–1.80)***
Diagnosed HF	3.39 (2.97–3.86)***	3.04 (2.62–3.53)***	3.01 (2.58–3.50)***
Diagnosed HF + LD use	3.96 (3.63–4.32)***	3.18 (2.85–3.54)***	3.00 (2.65–3.40)***
Ischaemic stroke^Δ^			
No HF or LD use	Referent	Referent	Referent
Isolated LD use	0.99 (0.94–1.04)	0.86 (0.81–0.91)***	0.88 (0.83–0.93)***
Diagnosed HF	0.98 (0.90–1.07)	1.00 (0.90–1.10)	1.02 (0.92–1.13)
Diagnosed HF + LD use	0.81 (0.76–0.86)***	0.72 (0.68–0.78)***	0.78 (0.72–0.84)***

*HF* heart failure, *LD* loop-diuretic

*p < 0.05, **p < 0.005, ***p < 0.0005

^Δ^Hazard ratios (95% CI) are derived using the method of Fine and Gray, adjusting for death as a competing risk

^a^Model 1 is the unadjusted analysis

^b^Model 2 adjusts for age, gender, systolic blood pressure, BMI, smoking status, and comorbidities at AF diagnosis: COPD, diabetes, hypertension, previous MI, previous stroke/TIA

^c^Model 3 adjusts for variables in Model 2 + medication use at AF diagnosis (ACEI/ARB, beta-blocker, MRA, digoxin, anticoagulant, antiplatelet, antiarrhythmic drug, non-dihydropyridine CCB, and statin use)

In gender-stratified analyses, survival after the onset of AF was worse for women than men (Additional file 2: Supplemental Figure 4) with evidence of effect modification by gender in the fully adjusted model (P_interaction_ = 0.016). This appeared to be driven by a greater risk of all-cause mortality in women (HR 1.39, 95% CI 1.27–1.53, p < 0.0005) versus men (HR 1.30 95% CI 1.20–1.40, p < 0.0005) with diagnosed HF and no loop-diuretic use. Interestingly, while mortality was higher in patients with isolated loop-diuretic use versus those without HF or loop-diuretic use (HR 1.42, 95% CI 1.37–1.47, p < 0.0005), the adjusted hazard ratio was lower in women (HR 1.38, 95% CI 1.31–1.44, p < 0.0005) than men (HR 1.47, 95% CI 1.40–1.54, p < 0.0005), after adjusting for the same set of confounding factors.

Age-standardised rates of unplanned HF hospitalisation and stroke were greater in women than men with HF (Additional file 2: Supplemental Table 4). However, there was no significant effect modification by gender in fully adjusted analyses for either unplanned HF hospitalisation (P_interaction_ 0.66) or stroke (P_interaction_ 0.25). The disproportionately high age-standardised rate of stroke observed in women with HF and loop-diuretic use was due to a single stroke event in a young person.

### Sensitivity analyses

When patients without at least four prescriptions for loop-diuretic agent(s) within 5 years were re-classified as having ‘no’ loop-diuretic use, there was little difference to the outcomes observed (Additional file 2: Supplemental Table 5). Similarly, when the analysis was restricted to patients with no documented valve disease (n = 109,844), there was no change in the observed trends (Additional file 2: Supplemental Table 6). Removal of the 100-day censoring period led to an increased risk of unplanned hospitalisation amongst patients with diagnosed HF, attributable both to a longer follow-up and to a higher mortality rate amongst individuals with a short follow-up duration (Additional file 2: Supplemental Tables 7 and 8). Increasing the censoring period from 100 days to 1 year (Additional file 2: Supplemental Table 9) and excluding loop-diuretic prescriptions issued within 12 months of death (Additional file 2: Supplemental Table 10) had no significant effects on the associations observed. A sensitivity analysis in patients without a BMI record showed similar estimated associations between isolated loop-diuretic use and outcomes (Additional file 2: Supplemental Table 11). When patients with stage 3–4 CKD were also considered as having a possible non-HF indication for loop-diuretic use (in addition to stage 5 and renal replacement therapy in the main analysis), this resulted in re-classification of 1257 individuals. The effect estimates remained robust with this sensitivity analysis (Additional file 2: Supplemental Table 12). Finally, we analysed the risks associated with isolated loop-diuretic use or HF diagnosis occurring at any time during follow-up, as time-dependent variables. With this approach, risk estimates remained broadly similar to the main analysis where participants were categorised according to their baseline exposure status, although the risk of unplanned HF hospitalisation was marginally greater for patients with isolated loop-diuretic use at any time and the association with ischaemic stroke was no longer statistically significant for this group (Additional file 2: Supplemental Table 13).

## Discussion

In this large, nationally representative cohort study of individuals with incident AF, 18% patients were prescribed loop-diuretic agents with no recorded diagnosis of HF and subsequently experienced a high risk of HF hospitalisation and death. Isolated loop-diuretic use was more common with increasing age and more common in women than men in every age-category. Importantly, one third of patients with a new diagnosis of HF (after AF onset) had an earlier record of ‘isolated’ loop-diuretic use, often for several months or years before HF or AF diagnosis. Taken together, these findings imply that the true prevalence of HF is substantially underestimated amongst patients with AF, particularly in women, and isolated loop-diuretic therapy may be a useful marker in primary care of unrecognised or impending HF, as well as a poor prognostic sign.

### Isolated loop-diuretic use in AF

Fluid retention and congestion are recognised hallmarks of HF syndromes, and loop-diuretics are frequently needed to achieve diuresis and symptom relief in this setting. Although a number of non-HF indications for loop-diuretics exist, and some degree of overlap with other chronic conditions invariably occurs, the observed similarities in baseline characteristics, including comorbidity burden, between patients with isolated loop-diuretic use and diagnosed HF, their increased risk of future HF hospitalisation, and comparable mortality risk to diagnosed HF, support at least a reasonable likelihood of unrecognised HF in a significant proportion of these individuals.

Under-recognition of HF in AF has been reported in older people [5], in hospitalised cohorts [17], and in clinical trials [18]. Furthermore, many individuals labelled as having ‘lone’ AF exhibit cardiac structural and functional abnormalities [19, 20], which are compatible with early or mild (stage B) HF. While existing AF guidelines recommend comprehensive evaluation of patients with a new presentation of AF, HF may also develop several months or years after initial AF diagnosis. In this scenario, repeat prescriptions of loop-diuretic agents may highlight individuals in primary care who should be reassessed, clinically, by cardiac imaging or measurement of natriuretic peptides.

The disproportionate number of women, high rates of hypertension, obesity, and lower rates of ischaemic heart disease seen in patients with isolated loop-diuretic use, resemble patient characteristics associated with HF and preserved (HFpEF) or mildly reduced EF (HFmrEF), more closely than HF with reduced EF (HFrEF), in population-based studies, and may suggest a predominance of these HF phenotypes in this AF subgroup. There is often a greater level of diagnostic uncertainty associated with HFpEF and HFmrEF, for which specialist evaluation may be required (e.g., exercise testing or invasive cardiac catheterisation). Further research is needed to identify whether targeted evaluation and enhanced clinical surveillance of patients with AF and isolated loop-diuretic use may lead to earlier and more comprehensive identification of these HF classifications. In addition, a subset of these patients had HF diagnosed during an emergency hospitalisation for acute decompensated HF. It is plausible that some of these admissions could be avoided with earlier HF diagnosis and initiation of treatment in primary or secondary outpatient care.

We cannot exclude the possibility that loop-diuretic agents themselves may have a detrimental effect, via potential electrolyte disturbances or neuro-endocrine activation, that may precipitate HF progression or adverse outcomes. Loop-diuretic therapy has additionally been associated with an increase in mortality in a Swedish cohort study of patients with AF and hypertension (n = 5602) [21]. In the current study, patients with isolated loop-diuretic use were, on average, less likely to receive prognostically beneficial treatments (ACEIs, ARBs, and MRAs) than patients with known HF [4], even though such therapies are also recommended for the treatment of hypertension (without HF) in AF [21, 22], which was equally prevalent. Thus, despite inexact specificity, repeat prescriptions of loop-diuretic agents in patients with AF represent a valuable opportunity to initiate or optimise prognostically beneficial therapy both for HF and HF-precursor conditions, or to stop potentially unnecessary prolonged diuretic use, wherever possible.

Unexpectedly, baseline prescription of loop-diuretics, in the presence or absence of a diagnosis of HF was associated with a lower risk of stroke in our analysis. This association was attenuated when isolated loop-diuretic use at any time during follow-up was examined, suggesting an element of immortal time bias. However, the apparent protective effect of HF with diuretic use persisted. This contrasts with previous studies where a convincing positive association [23] has been demonstrated between HF and stroke, even in the absence of AF [24]. Although we comprehensively adjusted for baseline medication use between groups, residual confounding may explain this result if, for example, loop-diuretic use was associated with greater compliance or lower discontinuation of cardioprotective medications such as statins or a lower time out of therapeutic range for anticoagulation, due to more frequent blood tests and assessment by healthcare professionals. That said, patients with HF who were not prescribed loop-diuretics use would also likely be under close surveillance but did not display a reduced risk of stroke. Patients treated with loop-diuretics also did not have a lower blood pressure that might have explained a difference in stroke rates. Thus, our finding of an apparent protective effect of loop-diuretic use on the risk of stroke requires further examination.

### Gender differences in AF outcomes

The ESC guidelines for AF stipulate a need for the ‘identification and resolution of sex-specific barriers to implementation of guideline-recommended treatments for AF’ [22]. Previous reports suggest that women with AF experience, on average, more symptoms, greater functional impairment, and poorer quality of life, than men [25–27], though data regarding sex differences in prognosis are conflicting [25, 27, 28]. In our large population cohort, women with AF had a worse prognosis than men, in particular women with AF and HF without congestion. Women in our cohort were older than men at the time of AF diagnosis and more commonly hypertensive. However, women were also less likely to have ischaemic heart disease or cancer. Fewer women were smokers, and on average, women had a lower BMI. Additionally, prescription rates of several medicines were either greater (beta-blockers, digoxin) for women, or similar (ACEI or ARB) between men and women, except anticoagulants which were less commonly prescribed to women. Thus, differences in characteristics do not provide a clear explanation for poorer outcomes for women with AF.

Our analysis raises another consideration: potential disparity in HF ascertainment between men and women with AF. More women had isolated loop-diuretic use than men, particularly older women. The median time between initiation of diuretics and a diagnosis of HF was also 1.5 years longer for women compared to men for those prescribed a loop-diuretic prior to a diagnosis of HF. This may reflect later detection of HF in women in primary care and may explain why more women than men received their HF diagnosis following an emergency hospitalisation. The reason why women with a diagnosis of HF who were not prescribed loop-diuretics had a higher mortality risk than men is unclear. Interestingly, when we combined isolated loop-diuretic use and diagnosed HF (i.e., ostensibly including all ‘possible’ HF in a single category), the median time between AF detection and ‘first’ HF presentation, as well as survival rates, became similar for men and women, suggesting under-recognition and under-diagnosis of HF in women with AF, rather than sex-specific (i.e., biological) differences in HF susceptibility or prognosis.

Potential sex differences in HF phenotype may be a relevant consideration. Our identification of a lower risk for all-cause mortality amongst the subset of women with isolated loop-diuretic use versus men, after adjusting for the same set of confounders, could suggest a higher proportion of HFpEF in women with AF, which has a somewhat better prognosis than HFrEF. In population-based studies, women reportedly have a higher [29] or similar [30] risk of developing HFpEF than men, but a lower risk of developing HFrEF. Interestingly, in the Renal and Vascular Endstage Disease (PREVEND) cohort, AF was identified as a sex-specific risk factor for HF, with AF conferring an increased risk of HFpEF in women but not in men [29]. Pathophysiological differences such as more concentric (versus eccentric) remodelling [31], greater aortic stiffness [32], impaired cardiovascular coupling [33], or higher heart rates [34] may be reasons for more clinically manifest HF in women compared to men with AF and preserved LV ejection fraction. However, this may also have been a chance finding and requires validation. Even though evidence-based treatment strategies are currently lacking for HFpEF, improved detection and accurate categorisation of HFpEF in women and men in primary care would encourage close monitoring of volume status, which can lead to symptomatic improvement and possibly avert hospitalisation [35], as well as minimise the use (and associated side effects) of ineffective therapies, such as those for HFrEF or bronchodilators that may be prescribed for symptoms falsely attributed to lung disease [36]. It would also identify eligible individuals for recruitment into research studies to test new strategies to improve their care.

### Strengths and limitations

Strengths of our study include the large and nationally representative cohort, allowing us to retain power for multiple sensitivity analyses, and comprehensive data regarding prescriptions. Loop-diuretic prescriptions identify the subset of HF patients who have fluid congestion, which will still underestimate the true overall prevalence of HF. Equally, as previously acknowledged, not all patients who receive diuretics do so for HF. Our stipulation of long-term loop-diuretic therapy and exclusion of common non-HF-related reasons improve the specificity of isolated loop-diuretic therapy as a potential surrogate marker of HF.

It is recognised that the quality of coding in primary care can be variable [37]. However, codes used in this study pertaining to HF and AF that are financially incentivised by schemes, such as the UK’s Quality and Outcomes Framework, are more likely to be complete [38]. Additional codes for covariates were either validated (e.g., COPD [39]) or identified and checked by two physician specialists. We did not have information regarding medication adherence, and adequacy of heart rate control or use of rhythm control procedures, which may have been sources of residual confounding of hospitalisation and survival rates. We applied a censoring period of 100 days when ascertaining outcomes to allow time for a diagnosis of HF and repeat prescriptions of loop-diuretics to be recorded (in primary care and between primary and secondary care). When we eliminated this censor period, there was a high mortality soon after AF diagnosis, particularly for patients with diagnosed HF. When we imposed a longer censoring period, this had little impact on the association between possible or diagnosed HF and all-cause mortality, suggesting that HF was not merely a pre-terminal event. For our main analyses, participants were categorised according to their baseline exposure status (i.e., whether they had evidence of HF or isolated loop-diuretic use around or before the time of AF diagnosis). We selected this approach to examine the importance of possible undiagnosed HF at the clinical interaction where AF is first diagnosed. Although this may underestimate the risks associated with exposure over a patient’s lifetime, the overall trends remained similar when exposures were defined using a time-dependent approach.

Amongst patient characteristics, BMI had a relatively high rate of missingness. Sensitivity analyses suggested that missing BMI data had little effect on our estimates, i.e., the results were similar in the cohort of patients with no BMI data available. We considered this approach more appropriate than multiple imputation, because underweight and overweight individuals, may be more likely to have their BMI recorded, thus contradicting the required missing at random assumption [40]. Other covariates studied had minimal (< 5%) missing data. Finally, although we controlled for patient characteristics and comorbidities, residual confounding due to unmeasured variables remains possible and causal inference cannot be made because of the nature of observational studies.

## Conclusions

A substantial proportion of patients with AF are prescribed loop-diuretic agents, even without a formal diagnosis of HF, and have an increased risk of HF hospitalisation and death. Thus, isolated long-term loop-diuretic use is not benign in this population. Targeted evaluation of these patients for unrecognised or early-stage HF may allow earlier intervention with guideline-recommended HF therapy and better outcomes, particularly amongst women with AF, in whom the prevalence of unrecognised HF appears to be the greatest.

## Supplementary Information

**Additional file 1.** Supplemental methods.

**Additional file 2.** Supplemental tables and figures.

## Data Availability

The authors declare that all data supporting the findings of this study are available within the article (and its supplemental information files). Individual participant data will not be made available due to confidentiality regulations.
