# Supplementary material for: Under-recognition of heart failure in patients with atrial fibrillation and the impact of gender: a UK population-based cohort study
Source: BMC Med. 2021 Aug 10;19:179. doi: 10.1186/s12916-021-02048-8 (PMC8353868; doi:10.1186/s12916-021-02048-8)
Supplement: Supplementary file 1 — Additional file 1. Supplemental methods. [file 12916_2021_2048_MOESM1_ESM.docx]

**Supplemental Methods**

|  | Page |
| --- | --- |
| 1. Identification of incident atrial fibrillation (AF) cohort and definition of AF | 2 |
| 1. Patient characteristics | 2 |
| 1. CHA_2_DS_2_-VASc score | 2 |
| 1. Identification of incident heart failure | 2 |
| 1. Read codes used to identify individuals with AF or flutter in primary care records | 4 |
| 1. ICD-10 codes used to identify individuals with AF or flutter in hospital discharge records | 5 |
| 1. Read codes used to identify individuals with heart failure in primary care records | 6 |
| 1. ICD-10 codes used to identify individuals with heart failure in hospital discharge records | 11 |

**Supplemental methods**

1. **Identification of incident atrial fibrillation (AF) cohort and definition of AF**

Integration of data from multiple electronic health record (EHR) sources has been shown to improve case detection of AF ^1^. Additionally, systematic review of medical diagnostic codes in CPRD, including AF, demonstrate a high validity of such codes, with a mean positive predictive value of 89% ^2^. Therefore, incident cases of AF or atrial flutter were first identified by records of specific Read codes in the CPRD (assigned as grade 1; **Supplemental Table 1**) or relevant International Classification of Diseases, Tenth Revision (ICD-10) codes at hospital discharge, with preference given to the earliest dated record rather than diagnostic source (**Supplemental Table 2**). Secondly, as the onset of AF often precedes its diagnosis, we incorporated an additional hierarchical step to identify the closest possible date to true AF onset. In cases where a less specific Read code, pertaining to an historical diagnosis of AF or AF monitoring (assigned as grade 2; **Supplemental Table 1**), was recorded earlier than a grade 1 Read code or ICD-10 code for AF/flutter, this date was selected as the index diagnosis. Cases with a grade 2 Read code, which was not subsequently corroborated by a grade 1 Read code in CPRD or relevant ICD-10 code, were not included in the final study cohort. Finally, although Read codes permit differentiation of AF and atrial flutter, ICD-10 terminology has one term for all categories. Since many patients with atrial flutter may additionally experience AF, it is commonplace for epidemiological analyses, as in the current study, to incorporate codes for atrial flutter as synonymous with AF ^1,3^.

1. **Patient characteristics**

Comorbid conditions, as recorded by a corresponding Read code or ICD-10 code, before and up to 3 months after index AF diagnosis were extracted. Prescriptions for other medication (besides loop diuretic agents) ±3 months of index AF diagnosis, including anticoagulation, were recorded as baseline treatment. Patients without a relevant record were assumed to be free from the condition or not taking the specified medication. For systolic and diastolic blood pressure measurements, smoking status, and body mass index (BMI), the most recent value within 2 years of index AF diagnosis was used. Individual CHA_2_DS_2_-VASc scores were also calculated to quantify stroke risk ^4^ (see below).

1. **CHA_2_DS_2_-VASc score**

The CHA_2_DS_2_-VASc score assigns 2 points for the presence of: age ≥75 years, previous stroke or transient ischaemic attack (TIA) and 1 point for the presence of congestive heart failure, hypertension, age 65-74 years inclusive, diabetes mellitus, vascular disease, and female gender ^4^. The values are summed and the overall score ranges from 0 to 9.

1. **Identification of incident heart failure (HF)**

Incident ‘diagnosed’ HF was defined as the date of first documentation of a HF diagnosis, either in primary care (Read code) or during any hospitalisation (ICD-10 discharge code, any position), >3 months after AF diagnosis. Patients without a recorded diagnosis of HF at AF diagnosis (±3 months) formed the referent population for ascertainment of incident HF diagnosis during long-term follow-up.

Incident ‘isolated’ loop-diuretic use was defined as the receipt of long-term loop diuretic therapy, in the absence of a documented diagnosis of HF or non-cardiac indication for their use, beginning from the first prescription date >3 months after AF diagnosis. To qualify as ‘long-term’ patients required three prescriptions of any loop-diuretic agent, administered within 100 days. Patients without diagnosed HF or isolated loop-diuretic use formed the referent population for ascertainment of incident isolated loop-diuretic use during long-term follow-up.

**E. Read codes** used to identify individuals with atrial fibrillation or flutter in primary care records

| Code Type | Medcode | Read Code | Description | Assigned Level* | # Incident cases (%) |
| --- | --- | --- | --- | --- | --- |
| READ | 1664 | G573000 | atrial fibrillation | 1 | 52,755 |
| READ | 2212 | G573.00 | atrial fibrillation and flutter | 1 | 22,104 |
| READ | 1268 | G573200 | paroxysmal atrial fibrillation | 1 | 11,502 |
| READ | 1757 | G573100 | atrial flutter | 1 | 2,807 |
| READ | 3757 | 3272 | ecg: atrial fibrillation | 1 | 1302 |
| READ | 6771 | 3273 | ecg: atrial flutter | 1 | 211 |
| READ | 23437 | G573z00 | atrial fibrillation and flutter nos | 1 | 119 |
| READ | 96076 | G573500 | persistent atrial fibrillation | 1 | 64 |
| READ | 107472 | G573600 | paroxysmal atrial flutter | 1 | 46 |
| READ | 96277 | G573400 | permanent atrial fibrillation | 1 | 33 |
| READ | 45773 | 6A9..00 | atrial fibrillation annual review | 1 | 28 |
| READ | 35127 | G573300 | non-rheumatic atrial fibrillation | 1 | 11 |
| READ | 9479 | 7936A00 | implant intravenous pacemaker for atrial fibrillation | 1 | 8 |
| READ | 92361 | 793M000 | Perc translum ablat pulmon vein to lft atrium conduct system | 1 | 7 |
| READ | 6345 | 14AN.00 | h/o: atrial fibrillation | 2 | 293 |
| READ | 18746 | 662S.00 | atrial fibrillation monitoring | 2 | 191 |
| READ | 93460 | 14AR.00 | history of atrial flutter | 2 | 54 |
| READ | 57832 | 9Os..00 | atrial fibrillation monitoring administration | 2 | 22 |
| READ | 39114 | 9hF1.00 | excepted from atrial fibrillation qual indic: inform dissent | 2 | 9 |
| READ | 90187 | 9Os0.00 | atrial fibrillation monitoring first letter | 2 | 6 |
| READ | 90188 | 9Os1.00 | atrial fibrillation monitoring second letter | 2 | 1 |
| READ | 105554 | 8CMW200 | atrial fibrillation care pathway | 2 | 1 |
| READ | 63350 | 9hF..00 | exception reporting: atrial fibrillation quality indicators | 2 | 0 |
| READ | 90189 | 9Os2.00 | atrial fibrillation monitoring third letter | 2 | 0 |
| READ | 90190 | 9Os3.00 | atrial fibrillation monitoring verbal invite | 2 | 0 |
| READ | 90191 | 9Os4.00 | atrial fibrillation monitoring telephone invite | 2 | 0 |
| Total Incident Cases | | | | | 91,574 |

* Level 2 codes only used to define AF incidence when a (later) corroborating level 1 or ICD-10 code was available. Where multiple codes where used in a single patient, the earliest code was selected.

**F.** ICD-10 codes used to identify individuals with atrial fibrillation or flutter in hospital discharge records

| Code Type | icd | Description | # Incident Cases (%) |
| --- | --- | --- | --- |
| ICD | I48 | Atrial fibrillation and flutter (incorporates all codes below) | 32,682 (100.0) |
| ICD | I48.0 | Paroxysmal atrial fibrillation | - |
| ICD | I48.1 | Persistent atrial fibrillation | - |
| ICD | I48.2 | Chronic atrial fibrillation | - |
| ICD | I48.3 | Typical atrial flutter | - |
| ICD | I48.4 | Atypical atrial flutter | - |
| ICD | I48.9 | Unspecified atrial fibrillation and atrial flutter | - |
| ICD | I48.91 | Unspecified atrial fibrillation | - |
| ICD | I48.92 | Unspecified atrial flutter | - |
| Total Incident Cases | | | 32,682 |

**G.** Read codes used to identify individuals with heart failure in primary care records

| Code Type | Medcode | Read Code | Description | # Prevalent cases (%)* | # Incident cases (%) |
| --- | --- | --- | --- | --- | --- |
| READ | 242 | 7901000 | Allotransplantation of heart NEC | - | - |
| READ | 250 | 7900 | Transplantation of heart and lung | 2 (0.0) | - |
| READ | 398 | G580.00 | Congestive heart failure | 1,840 (14.1) | 915 (13.1) |
| READ | 884 | G581.00 | Left ventricular failure | 2,955 (22.7) | 1,061 (15.2) |
| READ | 1223 | G58..11 | Cardiac failure | 238 (1.8) | 139 (2.0) |
| READ | 2062 | G58..00 | Heart failure | 2,387 (18.3) | 1,574 (22.6) |
| READ | 2906 | G580.11 | Congestive cardiac failure | 2,080 (15.9) | 1,140 (16.4) |
| READ | 3204 | G55..00 | Cardiomyopathy | 316 (2.4) | 99 (1.4) |
| READ | 3499 | G554300 | Hypertrophic non-obstructive cardiomyopathy | 48 (0.4) | 10 (0.1) |
| READ | 4024 | G58z.00 | Heart failure NOS | 112 (0.9) | 53 (0.8) |
| READ | 4438 | 7901 | Other transplantation of heart | 2 (0.0) | - |
| READ | 4915 | G555.00 | Alcoholic cardiomyopathy | 15 (0.1) | 5 (0.1) |
| READ | 5141 | G554000 | Congestive cardiomyopathy | 5 (0.0) | 1 (0.0) |
| READ | 5255 | G581000 | Acute left ventricular failure | 34 (0.3) | 14 (0.2) |
| READ | 5695 | G41z.11 | chronic cor pulmonale | 71 (0.5) | 36 (0.5) |
| READ | 5942 | G581.13 | Impaired left ventricular function | 390 (3.0) | 222 (3.2) |
| READ | 7251 | 33BA.00 | Impaired left ventricular function | 302 (2.3) | 150 (2.2) |
| READ | 7320 | G343.00 | Ischaemic cardiomyopathy | 13 (0.1) | 12 (0.2) |
| READ | 7535 | G554400 | Primary dilated cardiomyopathy | 64 (0.5) | 24 (0.3) |
| READ | 8010 | G551.00 | Hypertrophic obstructive cardiomyopathy | 92 (0.7) | 12 (0.2) |
| READ | 8966 | G5yy900 | Left ventricular systolic dysfunction | 747 (5.7) | 474 (6.8) |
| READ | 9384 | ZV42100 | [V]Heart transplanted | - | - |
| READ | 9402 | G55y.11 | Secondary dilated cardiomyopathy | 18 (0.1) | 4 (0.1) |
| READ | 9524 | G580.14 | Biventricular failure | 60 (0.5) | 28 (0.4) |
| READ | 9913 | 1O1..00 | Heart failure confirmed | 199 (1.5) | 168 (2.4) |
| READ | 10079 | G580.12 | Right heart failure | 37 (0.3) | 56 (0.8) |
| READ | 10154 | G580.13 | right ventricular failure | 19 (0.2) | 8 (0.1) |
| READ | 11284 | 585f.00 | Echocardiogram shows left ventricular systolic dysfunction | 254 (2.0) | 136 (2.0) |
| READ | 11351 | 585g.00 | Echo shows LVDD | 57 (0.4) | 23 (0.3) |
| READ | 11424 | G580300 | Compensated cardiac failure | 3 (0.0) | 5 (0.1) |
| READ | 12550 | G5yyA00 | Left ventricular diastolic dysfunction | 116 (0.9) | 53 (0.8) |
| READ | 13189 | 662g.00 | New York Heart Association classification - class II | 52 (0.4) | 66 (1.0) |
| READ | 16383 | 1O1..00 | Heart failure confirmed | 17 (0.1) | - |
| READ | 17278 | G58z.12 | Cardiac failure NOS | 21 (0.2) | 10 (0.1) |
| READ | 17851 | 8HBE.00 | Heart failure follow-up | 65 (0.5) | 82 (1.2) |
| READ | 18853 | 662f.00 | New York Heart Association classification - class I | 24 (0.2) | 32 (0.5) |
| READ | 19066 | 662h.00 | New York Heart Association classification - class III | 22 (0.2) | 28 (0.4) |
| READ | 20324 | R2y1000 | [D]Cardiorespiratory failure | - | 7 (0.1) |
| READ | 21837 | G232.00 | hypertensive heart&renal dis wth (congestive) heart failure | - | 1 (0.0) |
| READ | 21852 | G554200 | Familial cardiomyopathy | 4 (0.0) | - |
| READ | 22262 | G1yz100 | Rheumatic left ventricular failure | 3 (0.0) | - |
| READ | 22993 | G55z.00 | Cardiomyopathy NOS | 30 (0.2) | 7 (0.1) |
| READ | 23707 | G580000 | Acute congestive heart failure | 15 (0.1) | 10 (0.1) |
| READ | 24185 | R055100 | [D]Cardiogenic shock | 19 (0.2) | 9 (0.1) |
| READ | 27679 | SP08500 | Heart-lung transplant failure and rejection | - | - |
| READ | 27683 | G558100 | Cardiomyopathy in myotonic dystrophy | 2 (0.0) | - |
| READ | 27884 | G580200 | Decompensated cardiac failure | 41 (0.3) | 25 (0.4) |
| READ | 27964 | G582.00 | Acute heart failure | 11 (0.1) | 9 (0.1) |
| READ | 30667 | G557000 | Amyloid heart disease | 2 (0.0) | 3 (0.0) |
| READ | 30779 | 662W.00 | Heart failure annual review | 27 (0.2) | 34 (0.5) |
| READ | 32671 | G580100 | Chronic congestive heart failure | 25 (0.2) | 15 (0.2) |
| READ | 32898 | 8H2S.00 | admit heart failure emergency | 8 (0.1) | 7 (0.1) |
| READ | 32945 | 8CL3.00 | Heart failure care plan discussed with patient | 7 (0.1) | 11 (0.2) |
| READ | 34065 | G41y000 | Secondary pulmonary hypertension | 77 (0.6) | 53 (0.8) |
| READ | 36960 | ZV59600 | [V]Heart and lungs transplant status | - | 1 (0.0) |
| READ | 40834 | G554z00 | Other primary cardiomyopathy NOS | 1 (0.0) | - |
| READ | 41488 | G554100 | Constrictive cardiomyopathy | 1 (0.0) | - |
| READ | 41495 | 7901z00 | Other transplantation of heart NOS | - | - |
| READ | 42043 | G55y.00 | Secondary cardiomyopathy NOS | 1 (0.0) | - |
| READ | 47484 | SP08400 | Heart transplant failure and rejection | - | - |
| READ | 51214 | 662i.00 | New York Heart Association classification - class IV | 2 (0.0) | 2 (0.0) |
| READ | 52127 | G211100 | benign hypertensive heart disease with ccf | - | - |
| READ | 53626 | 7900000 | Allotransplantation of heart and lung | - | - |
| READ | 55850 | G558.00 | Cardiomyopathy in disease EC | - | - |
| READ | 56392 | 7937900 | Implantation of biventricular cardiac pacemaker system | 1 (0.0) | 4 (0.1) |
| READ | 57306 | G554.00 | Other primary cardiomyopathies | - | - |
| READ | 57987 | G234.00 | Hyperten heart&renal dis+both(congestv)heart and renal fail | - | - |
| READ | 58529 | 7901200 | Implantation of prosthetic heart | - | - |
| READ | 58938 | G55y000 | Cardiomyopathy due to drugs and other external agents | 1 (0.0) | - |
| READ | 59140 | G532100 | Pick's disease of heart | - | - |
| READ | 61073 | 7900z00 | Transplantation of heart and lung NOS | - | - |
| READ | 62718 | G21z100 | Hypertensive heart disease NOS with CCF | 2 (0.0) | 1 (0.0) |
| READ | 63548 | SP11200 | Cardiorespiratory failure as a complication of care | - | - |
| READ | 64438 | TB00000 | Heart transplant with complication, without blame | - | - |
| READ | 64673 | G557z00 | Nutritional and metabolic cardiomyopathy NOS | - | - |
| READ | 64837 | G558200 | Dystrophic cardiomyopathy | - | - |
| READ | 65756 | 7933 | Transluminal heart assist operations | - | - |
| READ | 66306 | SP11111 | Heart failure as a complication of care | - | - |
| READ | 66881 | 7933400 | Implantation of ventricular assist device | - | - |
| READ | 68682 | SP11100 | Cardiac insufficiency as a complication of care | - | - |
| READ | 68685 | G552.00 | Obscure African cardiomyopathy | - | - |
| READ | 68766 | G554011 | Congestive obstructive cardiomyopathy | - | - |
| READ | 69734 | 7901y00 | Other specified other transplantation of heart | - | - |
| READ | 69836 | ZV43200 | [V]Has artificial heart | - | - |
| READ | 70648 | Gyu5M00 | [X]Other hypertrophic cardiomyopathy | 5 (0.0) | 1 (0.0) |
| READ | 70855 | G558000 | Cardiomyopathy in Friedreich's ataxia | - | - |
| READ | 72668 | G210100 | malignant hypertensive heart disease with ccf | - | - |
| READ | 72769 | 7933z00 | Transluminal heart assist operation NOS | - | - |
| READ | 72939 | 7901100 | Xenotransplantation of heart | - | - |
| READ | 73604 | 7933700 | Implantation of biventricular assist device | - | - |
| READ | 82469 | 7936J00 | Implantation of intravenous biventricular cardiac pacemaker system | 1 (0.0) | 3 (0.0) |
| READ | 83502 | 662p.00 | Heart failure 6 month review | 2 (0.0) | 4 (0.1) |
| READ | 88739 | ZV45M00 | [V]Biventricular pacemaker in situ | 5 (0.0) | 13 (0.2) |
| READ | 90933 | 793L000 | Open implantation of ventricular assist device | - | - |
| READ | 90934 | 793L100 | Open removal of ventricular assist device | - | - |
| READ | 91886 | 7933300 | Transluminal removal of heart assist system | - | - |
| READ | 92266 | Gyu5N00 | [X]Other restrictive cardiomyopathy | - | - |
| READ | 93844 | 7901500 | Revision of transplantation of heart NEC | - | - |
| READ | 93947 | R055111 | [D]Heart shock | - | - |
| READ | 94870 | G580400 | congestive heart failure due to valvular disease | 2 (0.0) | 3 (0.0) |
| READ | 96799 | G5y4z00 | Post cardiac operation heart failure NOS | - | - |
| READ | 97443 | 793L.00 | Open heart assist operations | - | - |
| READ | 97617 | Gyu5P00 | [X]Other cardiomyopathies | - | - |
| READ | 97780 | G559.00 | Arrhythmogenic right ventricular cardiomyopathy | - | 1 (0.0) |
| READ | 98020 | G558z00 | Cardiomyopathy in diseases EC, NOS | - | - |
| READ | 98634 | Gyu5R00 | [X]Cardiomyopathy in metabolic diseases CE | - | - |
| READ | 100320 | 7933600 | Implantation of left ventricular assist device | - | - |
| READ | 100764 | 7933500 | Implantation of right ventricular assist device | - | - |
| READ | 100966 | G557.00 | Nutritional and metabolic cardiomyopathies | - | - |
| READ | 101015 | G554500 | Takotsubo cardiomyopathy | 2 (0.0) | 2 (0.0) |
| READ | 101137 | G583.11 | HFNEF - heart failure with normal ejection fraction | 5 (0.0) | 4 (0.1) |
| READ | 101138 | G583.00 | Heart failure with normal ejection fraction | 21 (0.2) | 22 (0.3) |
| READ | 102955 | G55A.00 | Tachycardiomyopathy | 1 (0.0) | 2 (0.0) |
| READ | 103732 | 8CMK.00 | Has heart failure management plan | - | 7 (0.1) |
| READ | 104275 | G584.00 | Right ventricular failure | 6 (0.1) | 13 (0.2) |
| READ | 104529 | G55A.11 | Tachycardia-induced cardiomyopathy | 1 (0.0) | - |
| READ | 104658 | G554511 | Stress cardiomyopathy | - | - |
| READ | 105542 | 8CeC.00 | preferred place of care for next exacerbation heart failure | - | - |
| READ | 105651 | G558400 | Amyloid cardiomyopathy | - | - |
| READ | 105798 | G557011 | Cardiac amyloidosis | - | - |
| READ | 106008 | 8CMW800 | Heart failure clinical pathway | - | 1 (0.0) |
| READ | 106198 | 661M500 | heart failure self-management plan agreed | - | 3 (0.0) |
| READ | 106897 | G583.12 | Heart failure with preserved ejection fraction | 6 (0.1) | 14 (0.2) |
| READ | 107397 | G5yyD00 | Left ventricular cardiac dysfunction | 31 (0.2) | 32 (0.5) |
| READ | 107416 | 7901300 | Piggyback transplantation of heart | - | - |
| READ | 108180 | G5yyE00 | Right ventricular systolic dysfunction | - | 7 (0.1) |
| READ | 111210 | 7933100 | Transluminal insertion of heart assist system NEC | - | - |
| READ | 111391 | 793Lz00 | Open heart assist operations NOS | - | - |
| READ | 111428 | 2JZ..00 | On optimal heart failure therapy | - | - |
| READ | 102955 | G55A.00 | Tachycardiomyopathy | - | - |
| READ | 103732 | 8CMK.00 | Has heart failure management plan | - | - |
| READ | 104275 | G584.00 | Right ventricular failure | - | - |
| READ | 104529 | G55A.11 | Tachycardia-induced cardiomyopathy | - | - |
| READ | 104658 | G554511 | Stress cardiomyopathy | - | - |
| READ | 105542 | 8CeC.00 | preferred place of care for next exacerbation heart failure | - | - |
| READ | 105651 | G558400 | Amyloid cardiomyopathy | - | - |
| READ | 105798 | G557011 | Cardiac amyloidosis | - | - |
| READ | 106008 | 8CMW800 | Heart failure clinical pathway | - | - |
| READ | 106198 | 661M500 | heart failure self-management plan agreed | - | - |
| READ | 106897 | G583.12 | Heart failure with preserved ejection fraction | - | - |
| READ | 107397 | G5yyD00 | Left ventricular cardiac dysfunction | - | - |
| READ | 107416 | 7901300 | Piggyback transplantation of heart | - | - |
| READ | 108180 | G5yyE00 | Right ventricular systolic dysfunction | - | - |
| READ | 111210 | 7933100 | Transluminal insertion of heart assist system NEC | - | - |
| READ | 111391 | 793Lz00 | Open heart assist operations NOS | - | - |
| READ | 111428 | 2JZ..00 | On optimal heart failure therapy | - | - |
| READ | 106008 | 8CMW800 | Heart failure clinical pathway | - | - |
| READ | 106198 | 661M500 | heart failure self-management plan agreed | - | - |
| READ | 106897 | G583.12 | Heart failure with preserved ejection fraction | - | - |
| READ | 107397 | G5yyD00 | Left ventricular cardiac dysfunction | - | - |
| READ | 107416 | 7901300 | Piggyback transplantation of heart | - | - |
| READ | 108180 | G5yyE00 | Right ventricular systolic dysfunction | - | - |
| READ | 111210 | 7933100 | Transluminal insertion of heart assist system NEC | - | - |
| READ | 111391 | 793Lz00 | Open heart assist operations NOS | - | - |
| READ | 111428 | 2JZ..00 | On optimal heart failure therapy | - | - |
|  |  |  | **Total # Cases** | 13,045 (100.0) | 6,971 (100.0) |
|  |  |  | **Proportion of all HF cases (primary and secondary care)** | 59.2% | 57.2% |

*Refers to the earliest recorded HF-related diagnostic code. For patients with a concomitant Read code and ICD-10 code for HF, the latter was taken, and secondary care was assigned as the diagnostic location for HF.

**H.** ICD-10 codes used to identify individuals with heart failure in hospital discharge records

| Code Type | icd | Description | # Prevalent Cases (%) | # Incident Cases (%) |
| --- | --- | --- | --- | --- |
| ICD | I50.1 | Left ventricular failure, unspecified | 5,638 (63.0) | 2,964 (56.9) |
| ICD | I50.9 | Heart failure, unspecified | 2,068 (23.1) | 1,750 (33.6) |
| ICD | I42.0 | Dilated cardiomyopathy | 481 (5.4) | 151 (2.9) |
| ICD | I42.9 | Cardiomyopathy, unspecified. Secondary cardiomyopathy. Tachycardia induced cardiomyopathy. | 243 (2.7) | 111 (2.1) |
| ICD | I25.5 | Ischaemic cardiomyopathy | 140 (1.6) | 76 (1.5) |
| ICD | I11.0 | Hypertension with congestive heart failure | 109 (1.2) | 70 (1.3) |
| ICD | I42.1 | Obstructive hypertrophic cardiomyopathy | 87 (1.0) | 16 (0.3) |
| ICD | I42.2 | Other hypertrophic cardiomyopathy | 78 (0.9) | 28 (0.5) |
| ICD | I42.6 | Alcoholic cardiomyopathy | 52 (0.6) | 12 (0.2) |
| ICD | I42.8 | Other cardiomyopathies | 19 (0.2) | 11 (0.2) |
| ICD | I42.5 | Other restrictive cardiomyopathy | 13 (0.2) | 4 (0.1) |
| ICD | I13.0 | Hypertensive heart and chronic kidney disease with heart failure and stage 1 through stage 4 chronic kidney disease, or unspecified chronic kidney | 12 (0.1) | 4 (0.1) |
| ICD | I13.2 | Hypertensive heart and chronic kidney disease with heart failure and with stage 5 chronic kidney disease, or end stage renal disease | 11 (0.1) | 13 (0.2) |
| ICD | I42.7 | Cardiomyopathy due to drug and external agent | 5 (0.1) | 1 (0.0) |
| ICD | I42 | Cardiomyopathy | - | - |
| ICD | I50 | Heart failure | - | - |
| ICD | I50.2 | Systolic (congestive) heart failure | - | - |
| ICD | I50.20 | Unspecified systolic (congestive) heart failure | - | - |
| ICD | I50.21 | Acute systolic (congestive) heart failure | - | - |
| ICD | I50.22 | Chronic systolic (congestive) heart failure | - | - |
| ICD | I50.23 | Acute on chronic systolic (congestive) heart failure | - | - |
| ICD | I50.3 | Diastolic (congestive) heart failure | - | - |
| ICD | I50.30 | Unspecified diastolic (congestive) heart failure | - | - |
| ICD | I50.31 | Acute diastolic (congestive) heart failure | - | - |
| ICD | I50.32 | Chronic diastolic (congestive) heart failure | - | - |
| ICD | I50.33 | Acute on chronic diastolic (congestive) heart failure | - | - |
| ICD | I50.4 | Combined systolic (congestive) and diastolic (congestive) heart failure | - | - |
| ICD | I50.40 | Unspecified combined systolic (congestive) and diastolic (congestive) heart failure | - | - |
| ICD | I50.41 | Acute combined systolic (congestive) and diastolic (congestive) heart failure | - | - |
| ICD | I50.42 | Chronic combined systolic (congestive) and diastolic (congestive) heart failure | - | - |
| ICD | I50.43 | Acute on chronic combined systolic (congestive) and diastolic (congestive) heart failure | - | - |
| ICD | I50.8 | Other heart failure | - | - |
| ICD | I50.81 | Right heart failure | - | - |
| ICD | I50.810 | Right heart failure unspecified | - | - |
| ICD | I50.811 | Acute right heart failure | - | - |
| ICD | I50.812 | Chronic right heart failure | - | - |
| ICD | I50.813 | Acute on chronic right heart failure | - | - |
| ICD | I50.814 | Acute on chronic right heart failure due to left heart failure | - | - |
| ICD | I50.82 | Biventricular heart failure | - | - |
| ICD | I50.83 | High output heart failure | - | - |
| ICD | I50.84 | End stage heart failure | - | - |
| ICD | I50.89 | Other heart failure | - | - |
| Total # Cases | | | 8,956 (100.0) | 5,211 (100.0) |
| Proportion of all HF cases (primary and secondary care) | | | 40.7% | 42.8% |

**References**

1. Morley KI, Wallace J, Denaxas SC, et al. Defining disease phenotypes using national linked electronic health records: a case study of atrial fibrillation. *PLoS One* 2014; **9**(11): e110900.

2. Herrett E, Thomas SL, Schoonen WM, Smeeth L, Hall AJ. Validation and validity of diagnoses in the General Practice Research Database: a systematic review. *Br J Clin Pharmacol* 2010; **69**(1): 4-14.

3. Zakeri R, Chamberlain AM, Roger VL, Redfield MM. Temporal relationship and prognostic significance of atrial fibrillation in heart failure patients with preserved ejection fraction: a community-based study. *Circulation* 2013; **128**(10): 1085-93.

4. Lip GY, Nieuwlaat R, Pisters R, Lane DA, Crijns HJ. Refining clinical risk stratification for predicting stroke and thromboembolism in atrial fibrillation using a novel risk factor-based approach: the euro heart survey on atrial fibrillation. *Chest* 2010; **137**(2): 263-72.
