# Supplementary material for: Under-recognition of heart failure in patients with atrial fibrillation and the impact of gender: a UK population-based cohort study
Source: BMC Med. 2021 Aug 10;19:179. doi: 10.1186/s12916-021-02048-8 (PMC8353868; doi:10.1186/s12916-021-02048-8)
Supplement: Supplementary file 2 — Additional file 2. Supplemental tables and figures. [file 12916_2021_2048_MOESM2_ESM.docx]

**Supplemental Tables and Figures**

|  | | Page |
| --- | --- | --- |
| *Supplemental tables* | | |
| Table 1. | Crude incidence rates for heart failure diagnosis and isolated loop-diuretic use in patients with no prevalent heart failure or loop-diuretic use at AF diagnosis | 2 |
| Table 2. | Rates of adverse outcomes (>100 days) after AF diagnosis by baseline heart failure status. | 3 |
| Table 3. | Patient characteristics at AF diagnosis by gender. | 4 |
| Table 4. | Rates of adverse outcomes after AF diagnosis by gender and baseline heart failure status. | 6 |
| Table 5. | Risk of adverse outcomes after AF diagnosis, by baseline heart failure status.  *Patients with an isolated episode of heart failure classified as no heart failure.* | 7 |
| Table 6. | Risk of adverse outcomes after AF diagnosis, by baseline heart failure status.  *Including only patients with non-valvular AF* | 8 |
| Table 7. | Adverse outcomes after AF diagnosis by baseline heart failure status.  *Including outcomes occurring within 100-days of AF diagnosis.* | 9 |
| Table 8. | Risk of adverse outcomes after AF diagnosis, by baseline heart failure status.  *No censor period after AF diagnosis.* | 10 |
| Table 9. | Risk of adverse outcomes after AF diagnosis, by baseline heart failure status.  *Increasing the censor period after AF diagnosis to 1 year.* | 11 |
| Table 10. | Risk of adverse outcomes after AF diagnosis, by baseline heart failure status.  *Excluding loop-diuretic use in the last 12 months of life.* | 12 |
| Table 11. | Risk of adverse outcomes after AF diagnosis, by baseline heart failure status.  *Restricted to patients with no BMI measurement.* | 13 |
| Table 12. | Risk of adverse outcomes after AF diagnosis, by baseline heart failure status.  *Re-classifying patients with stage 3 and 4 chronic kidney disease (n=1,257) as having a non-cardiac indication for loop-diuretic therapy (in addition to those with stage 5 and renal replacement therapy).* | 14 |
| Table 13. | Risk of adverse outcomes after AF diagnosis by heart failure status at any time during follow-up*. Exposure categories defined using a time-dependent approach (i.e., isolated loop-diuretic use or HF diagnosis occurring at any time during follow-up).* | 15 |
| *Supplemental figures* | | |
| Figure 1. | Prevalence of diagnosed heart failure and isolated loop-diuretic use at AF diagnosis by study year. Includes heart failure diagnosed before [>3months] and concomitant with [±3months] AF diagnosis. | 17 |
| Figure 2. | Prevalence of diagnosed heart failure and isolated loop-diuretic use at AF diagnosis by gender. A. Women, B. Men. Includes HF diagnosed prior to [>3months] and concomitant with [±3months] AF diagnosis. | 18 |
| Figure 3. | Cumulative incidence of (A) diagnosed heart failure and (B) diagnosed heart failure combined with isolated loop-diuretic use, and the competing risk of death before heart failure, after AF diagnosis. Study cohort comprises patients without heart failure or loop-diuretic use at AF diagnosis. | 19 |
| Figure 4. | Survival after AF diagnosis, stratified by gender and baseline heart failure status. A. Diagnosed heart failure versus no heart failure. B. Diagnosed heart failure or isolated loop-diuretic use versus no heart failure. **excluding a 100-day censor period.* | 20 |

**Supplemental Table 1.** Crude incidence rates for heart failure diagnosis and isolated loop-diuretic use in patients with no prevalent heart failure or loop-diuretic use at AF diagnosis

| Outcome | Overall  n=124,256 | Women  n=58,222 (46·9%) | Men  n=66,034 (53·1%) | p-value (women vs men) | Incidence rate ratio (women/men), 95% CI,  p-value |
| --- | --- | --- | --- | --- | --- |
| Incident HF diagnosis^a^ |  |  |  |  |  |
| No. events /No. at risk (%) | 12,182 / 102,255 (11·9) | 5,586 / 48,361 (11·6) | 6,596 / 53,894 (12·2) |  |  |
| Patient-years at risk | 380,639·7 | 175,305·7 | 205,334·0 |  |  |
| Crude event rate  /100pt-yrs (95% CI) | 3·2  (3·1-3·3) | 3·2  (3·1-3·3) | 3·2  (3·1-3·3) |  | 0·99  (0·96-1·03)  p=0·656 |
| Standardised event rate^c^ /100pt-yrs (95% CI) | 1·7  (1·7-1·7) | 1·5  (1·5-1·5) | 1·8  (1·7-1·8) |  |  |
| Hospital HF diagnosis,  n (% of all HF) | 3,964  (32·5) | 1,900  (34·0) | 2,064  (31·3) | 0·001 |  |
| Incident HF diagnosis or isolated loop diuretic use^b^ | | | | | |
| No. events / No. at risk (%) | 17,307 / 79,930 (26·9) | 8,226 / 35,111 (23·4) | 9,081 / 44,819 (20·3) |  |  |
| Patient-years at risk | 285,185 | 119,840·9 | 165,344·1 |  |  |
| Crude event rate  /100pt-yrs (95% CI) | 6·1  (6·0-6·2) | 6·9  (6·7-7·0) | 5·5  (5·4-5·6) |  | 1·25  (1·21-1·29)  p=<0·00005 |
| Standardised event rate^c^ /100pt-yrs (95% CI) | 3·0  (3·0-3·1) | 3·1  (3·1-3·2) | 2·8  (2·8-2·9) |  |  |
| Time to diagnosed or possible HF from AF diagnosis, yrs | 2·1  (0·8-4·5) | 2·1  (0·8-4·4) | 2·1  (0·8-4·5) | 0·061 |  |

Data presented as median (LQ-UQ)

^a^ Study population includes all patients without prevalent HF at AF diagnosis

^b^ Study population includes all patients without prevalent HF *or* loop diuretic use at AF diagnosis.

^c^ Standardised according to the distribution of age and gender (overall analysis) or age alone (gender-stratified analyses) of the UK population in CPRD in 2011 (*de Jong RG, Gallagher AM, Herrett E, Masclee AA, Janssen-Heijnen ML, de Vries F. Comparability of the age and gender distribution of the UK Clinical Practice Research Datalink and the total Dutch population. Pharmacoepidemiol Drug Saf 2016; 25(12): 1460-4*).

**Supplemental Table 2.** Rates of adverse outcomes (>100 days) after AF diagnosis by baseline heart failure status

| Outcome | Total population  n=124,256 | Prevalent heart failure at AF diagnosis? | | | |
| --- | --- | --- | --- | --- | --- |
|  |  | **No**  **n=102,255** | | **Yes**  **n=22,001** | |
|  |  | **Treated with loop diuretic?** | | | |
|  |  | **No**  **n=79,930** | **Yes**  **n=22,235** | **No**  **n=6,082** | **Yes**  **n=15,919** |
| All-cause mortality |  |  |  |  |  |
| No. events (%) | 32,258 (26.0) | 16,084 (20.1) | 7,842 (35.3) | 1,608 (26.4) | 6,744 (42.4) |
| Person-years at risk | 444,575.7 | 306,586.7 | 71,736.3 | 19,864.4 | 46,388.3 |
| Crude event rate  per 100pt-yrs (95% CI^a^) | 7.3  (7.2-7.3) | 5.2  (5.2-5.3) | 10.9  (10.7-11.2) | 8.1  (7.7-8.5) | 14.5  (14.2-14.9) |
| Standardised event rate^b^  per 100pt-yrs (95%CI^a^) | 2.9  (2.9-2.9) | 2.3  (2.2-2.3) | 4.0  (3.9-4.1) | 4.1  (3.9-4.3) | 7.3  (7.1-7.5) |
| Emergency HF hospitalisation |  |  |  |  |  |
| No. events (%) | 3,064 (2.5) | 1,233 (1.5) | 648 (2.9) | 276 (4.5) | 907 (5.7) |
| Person-years at risk | 435,943.9 | 303,903.8 | 70,558.5 | 18,420.0 | 43,061.6 |
| Crude event rate  per 100pt-yrs (95% CI^a^) | 0.7  (0.7-0.7) | 0.4  (0.4-0.4) | 0.9  (0.9-1.0) | 1.5  (1.3-1.7) | 2.1  (2.0-2.2) |
| Standardised event rate^b^  per 100pt-yrs (95%CI^a^) | 0.4  (0.4-0.5) | 0.2  (0.2-0.3) | 0.9  (0.8-1.0) | 1.9  (1.7-2.2) | 2.3  (2.2-2.5) |
| Ischaemic stroke/TIA |  |  |  |  |  |
| No. events (%) | 11,584 (9.3) | 7,660 (9.6) | 2,128 (9.6) | 531 (8.7) | 1,265 (7.9) |
| Person-years at risk | 401,043.2 | 275,479.3 | 64,893.4 | 17,899.6 | 42,770.9 |
| Crude event rate  per 100pt-yrs (95% CI^a^) | 2.9  (2.8-2.9) | 2.8  (2.7-2.8) | 3.3  (3.1-3.4) | 3.0  (2.7-3.2) | 3.0  (2.8-3.1) |
| Standardised event rate^b^  per 100pt-yrs (95%CI^a^) | 1.4  (1.4-1.4) | 1.4  (1.4-1.4) | 1.6  (1.5-1.6) | 1.6  (1.5-1.7) | 2.2  (2.1-2.4) |

HF, heart failure; TIA, transient ischaemic attack

^a^Assuming a constant incidence rate over time.

^b^Standardised for the age and gender-distribution of the UK population in CPRD in 2011

**Supplemental Table 3. Patient characteristics at AF diagnosis by gender**

| Characteristic | Total population with incident AF  n=124,256 | Men  N=66,034 (53.1%) | Women  N=58,222 (46.9%) | p-value |
| --- | --- | --- | --- | --- |
| Age (y) | 76 (68, 83) | 73 (65, 81) | 79 (71, 85) | <0.0005 |
| Systolic blood pressure (mmHg)  Missing data, n (%) | 133 (120, 145)  1,459 (1.2) | 132 (120, 143)  869 (1.3) | 135 (121, 146)  590 (1.0) | <0.0005 |
| Diastolic blood pressure (mmHg)  Missing data, n (%) | 79 (70, 84)  1,459 (1.2) | 79 (70, 84)  869 (1.3) | 79 (70, 84)  590 (1.0) | 0.588 |
| BMI (kg/m^2^)  BMI category^b^, n (%)  Underweight  Normal  Overweight  Obese  Missing data | 28.1±6.2  2,647 (2.1)  26,909 (21.7)  33,401 (26.9)  30,765 (24.8)  30,534 (24.6) | 28.3±5.6  746 (1.1)  13,239 (20.0)  20,427 (30.9)  16,835 (25.5)  14,787 (22.4) | 27.9±6.8  1,901 (3.3)  13,670 (23.4)  12,974 (22.3)  13,930 (23.9)  15,747 (27.0) | <0.0005  <0.0005 |
| Smoking, n (%)  Current smovenomgt  ker  Ex-smoker  Total | 35,106 (28.3)  36,115 (29.1)  71,221 (57.3) | 21,537 (32.6)  44,325 (67.1) | 13,569 (23.3)  26,896 (46.2) | <0.005  <0.005  <0.005 |
| Comorbidities, n (%) |  |  |  |  |
| *Cardiovascular* |  |  |  |  |
| Hypertension | 81,137 (65.3) | 40,456 (61.3) | 40,681 (69.9) | <0.0005 |
| Ischaemic heart disease | 37,286 (30.0) | 21,823 (33.1) | 15,463 (26.6) | <0.0005 |
| Prior myocardial infarction | 15,208 (12.2) | 9,983 (15.1) | 5,225 (9.0) | <0.0005 |
| Stroke/TIA | 17,744 (14.3) | 9,011 (13.7) | 8,733 (15.0) | <0.0005 |
| Valvular heart disease | 14,412 (11.6) | 7,135 (10.8) | 7,277 (12.5) | <0.0005 |
| PPM / ICD | 3,008 (2.4) | 1,907 (2.9) | 1,101 (1.9) | <0.0005 |
| Peripheral artery disease | 10,367 (8.3) | 6,241 (9.5) | 4,126 (7.1) | <0.0005 |
| Dyslipidaemia | 32,477 (26.1) | 17,554 (26.6) | 14,923 (25.6) | <0.0005 |
| *Respiratory* |  |  |  |  |
| Asthma | 20,198 (16.3) | 9,851 (14.9) | 10,347 (17.8) | <0.0005 |
| COPD | 14,943 (12.0) | 8,500 (12.9) | 6,443 (11.1) | <0.0005 |
| Interstitial lung disease | 2,776 (2.2) | 1,534 (2.3) | 1,242 (2.1) | 0.024 |
| Obstructive sleep apnoea | 1,464 (1.2) | 1,212 (1.8) | 252 (0.4) | <0.0005 |
| *Renal* |  |  |  |  |
| End-stage kidney disease | 2,286 (1.8) | 1,258 (1.9) | 1,028 (1.8) | 0.068 |
| Nephrotic syndrome | 259 (0.2) | 166 (0.3) | 93 (0.2) | <0.0005 |
| *Other* |  |  |  |  |
| Diabetes | 20,684 (16.7) | 12,031 (18.2) | 8,653 (14.9) | <0.0005 |
| Chronic liver disease | 1,325 (1.1) | 745 (1.1) | 580 (1.0) | 0.024 |
| Malignancy (any)  Malignancy (top 4 causes)^c^ | 21,076 (17.0)  5,438 (4.4) | 11,700 (17.7)  3,126 (4.7) | 9,376 (16.1)  2,312 (4.0) | <0.0005  <0.0005 |
| Depression | 18,565 (14.9) | 7,301 (11.1) | 11,264 (19.4) | <0.0005 |
| CHA_2_DS_2_-VASc score | 3.3±1.7 | 2.7±1.7 | 4.0±1.5 | <0.0005 |
| Medication, n (%) |  |  |  |  |
| ACEI | 64,855 (52.2) | 34,904 (52.9) | 29,951 (51.4) | <0.0005 |
| ARB | 7,435 (6.0) | 3,371 (5.1) | 4,064 (7.0) | <0.0005 |
| ACEI or ARB | 72,290 (58.2) | 38,275 (58.0) | 34,015 (58.4) | 0.101 |
| ARNI | <5 (0.0) | <5 (0.0) | <5 (0.0) | - |
| MRA | 9,277 (7.5) | 4,991 (7.6) | 4,286 (7.4) | 0.188 |
| Antiarrhythmic drug^d^ | 11,195 (9.0) | 6,725 (10.2) | 4,470 (7.7) | <0.0005 |
| Beta-blocker | 81,591 (65.7) | 42,214 (63.9) | 39,377 (67.6) | <0.0005 |
| Digoxin | 30,309 (24.4) | 13,726 (20.8) | 16,583 (28.5) | <0.0005 |
| Dihydropyridine CCB | 50,994 (41.0) | 25,525 (38.7) | 25,469 (43.7) | <0.0005 |
| Non-dihydropyridine CCB | 16,588 (13.4) | 8,022 (12.2) | 8,566 (14.7) | <0.0005 |
| Anticoagulant therapy | 58,348 (47.0) | 32,762 (49.6) | 25,586 (44.0) | <0.0005 |
| Antiplatelet therapy | 86,018 (69.2) | 45,478 (68.9) | 40,540 (69.6) | 0.004 |
| Loop diuretic (non-cardiac indication) | 1,125 (0.9) | 569 (1.3) | 556 (1.6) | <0.0005 |
| Statin | 65,336 (52.6) | 36,591 (55.4) | 28,745 (49.4) | <0.0005 |

Data presented as mean (SD), median (LQ, UQ) or frequency (%) as appropriate.

BMI *denotes* body mass index; COPD, chronic obstructive pulmonary disease; ICD, implantable cardioverter defibrillator; PPM, permanent pacemaker; TIA, transient ischaemic attack.

^a^ Refers to loop diuretic therapy for a presumed cardiac indication. Patients on loop diuretic therapy with end-stage renal failure, chronic liver disease, or nephrotic syndrome (without a diagnosis of heart failure) were considered to have a non-cardiac indication and therefore included in the ‘no loop diuretic therapy’ category for this analysis. Patients with heart failure and loop diuretic use were assumed to have a cardiac indication.

^b^ BMI was categorised as underweight (<18.5 kg/m^2^), normal (18.5–24.9 kg/m^2^), overweight (25–29.9 kg/m^2^), and obese (≥30 kg/m^2^).

^c^ Aggregated number of cases of the four most common causes of cancer (lung, breast, bowel, and prostate) as reported by Cancer Research UK in 2015 (Cancer Research UK. Cancer incidence statistics. <https://www.cancerresearchuk.org/health-professional/cancer-statistics/incidence>; accessed Jun 02, 2021).

^d^ Excluding non-dihydropyridine calcium channel blockers

**Supplemental Table 4.**  Rates of adverse outcomes after AF diagnosis by gender and baseline heart failure (HF) status

| Outcome | Women  n=58,222 | | | | Men  n=66,034 | | | |
| --- | --- | --- | --- | --- | --- | --- | --- | --- |
|  | **No heart failure** | | **Diagnosed heart failure** | | **No heart failure** | | **Diagnosed heart failure** | |
|  | **No loop diuretic**  **n=35,111** | **Isolated loop diuretic use**  **n=13,250** | **No loop diuretic**  **n=2,407** | **Loop diuretic**  **n=7,454** | **No loop diuretic**  **n=44,819** | **Isolated loop diuretic use**  **n=9,075** | **No loop diuretic**  **n=3,675** | **Loop diuretic**  **n=8,465** |
| All-cause mortality |  |  |  |  |  |  |  |  |
| No. events (%) | 7,464 (21·2) | 4,548 (34·3) | 694 (28·8) | 3,190 (42·8) | 8,600 (19·1) | 3,294 (36·3) | 914 (24·9) | 3,554 (42·0) |
| Patient-years at risk | 130,640·4 | 42,317·5 | 7,144·9 | 20,869·6 | 175,946·3 | 29,418·8 | 12,719·5 | 25,518·7 |
| Crude event rate  per 100pt-yrs (95% CI) | 5·7  (5·6-5·8) | 10·7  (10·4-11·1) | 9·7  (9·0-10·4) | 15·3  (14·8-15·8) | 4·9  (4·8-5·0) | 11·2  (10·8-11·6) | 7·2  (6·7-7·7) | 13·9  (13·5-14·4) |
| Standardised event rate  per 100pt-yrs (95%CI^a^) | 2·3  (2·3-2·4) | 4·3  (4·2-4·5) | 3·7  (3·4-4·0) | 7·3  (7·1-7·6) | 2·1  (2·0-2·1) | 3·8  (3·7-3·9) | 3·8  (3·6-4·1) | 7·0  (6·8-7·2) |
| Unplanned HF hospitalisation |  |  |  |  |  |  |  |  |
| No. events (%) | 564 (1·6) | 367 (2·8) | 104 (4·3) | 419 (5·6) | 669 (1·5) | 281 (3·1) | 172 (4·7) | 488 (5·8) |
| Patient-years at risk | 129,460·9 | 41,613·9 | 6,561·4 | 19,403·7 | 177,442·9 | 28,944·6 | 11,858·6 | 23,657·9 |
| Crude event rate  per 100pt-yrs (95% CI) | 0·4  (0·4-0·5) | 0·9  (0·8-1·0) | 1·6  (1·3-1·9) | 2·2  (2·0-2·4) | 0·4  (0·3-0·4) | 1·0  (0·9-1·1) | 1·5  (1·2-1·7) | 2·1  (1·9-2·3) |
| Standardised event rate  per 100pt-yrs (95%CI^a^) | 0·2  (0·2-0·2) | 1·1  (0·9-1·2) | 2·5  (2·0-3·0) | 4·2  (3·8-4·7) | 0·2  (0·2-0·3) | 0·8  (0·7-0·9) | 1·8  (1·5-2·1) | 1·9  (1·7-2·0) |
| Stroke |  |  |  |  |  |  |  |  |
| No. events (%) | 3,652 (10·4) | 1,328 (10·0) | 229 (9·5) | 626 (8·4) | 4,008 (8·9) | 800 (8·8) | 302 (8·2) | 639 (7·5) |
| Patient-years at risk | 116,430·6 | 38,178·2 | 6,419·6 | 19,070·8 | 159,048·7 | 26,715·2 | 11,479·9 | 23,700·1 |
| Crude event rate  per 100pt-yrs (95% CI) | 3·1  (3·0-3·2) | 3·5  (3·3-3·7) | 3·6  (3·1-4·1) | 3·3  (3·0-3·5) | 2·5  (2·4-2·6) | 3·0  (2·8-3·2) | 2·6  (2·4-2·9) | 2·7  (2·5-2·9) |
| Standardised event rate  per 100pt-yrs (95%CI^a^) | 1·5  (1·4-1·5) | 1·6  (1·5-1·7) | 2·3  (2·0-2·6) | 10·4  (9·6-11·3) | 1·3  (1·2-1·3) | 1·5  (1·4-1·6) | 1·5  (1·3-1·6) | 1·5  (1·4-1·7) |

HF, heart failure

^a^The 95% CI estimates assume a constant incidence rate over time

**Supplemental Table 5. Risk of adverse outcomes after AF diagnosis, by baseline heart failure status.** *Patients with an isolated episode of heart failure re-classified as no heart failure.*

| Outcome | Model 1^a^ | Model 2^b^ | Model 3^c^ |
| --- | --- | --- | --- |
|  | **HR (95% CI)** | **HR (95% CI)** | **HR (95% CI)** |
| All-cause mortality |  |  |  |
| No HF or LD use | Referent | Referent | Referent |
| Isolated LD use | 2·08 (2·02-2·14)*** | 1·44 (1·40-1·49)*** | 1·40 (1·35-1·45)*** |
| Diagnosed HF | 1·69 (1·61-1·77)*** | 1·48 (1·40-1·56)*** | 1·43 (1·36-1·51)*** |
| Diagnosed HF + LD use | 2·70 (2·63-2·78)*** | 1·88 (1·81-1·95)*** | 1·71 (1·64-1·78)*** |
| Unplanned HF hospitalisation ^Δ^ |  |  |  |
| No HF or LD use | Referent | Referent | Referent |
| Isolated LD use | 1·94 (1·76-2·14)*** | 1·67 (1·48-1·88)*** | 1·64 (1·46-1·85)*** |
| Diagnosed HF | 3·32 (2·94-3·76)*** | 3·00 (2·59-3·42)*** | 2·92 (2·53-3·38)*** |
| Diagnosed HF + LD use | 3·96 (3·63-4·32)*** | 3·18 (2·85-3·54)*** | 3·01 (2·66-3·40)*** |
| Ischaemic stroke ^Δ^ | | | |
| No HF or LD use | Referent | Referent | Referent |
| Isolated LD use | 0·98 (0·93-1·03) | 0·85 (0·80-0·90)*** | 0·87 (0·82-0·93)*** |
| Diagnosed HF | 0·96 (0·88-1·04) | 0·96 (0·87-1·05) | 0·99 (0·90-1·09) |
| Diagnosed HF + LD use | 0·81 (0·76-0·86)*** | 0·72 (0·67-0·78)*** | 0·78 (0·72-0·84)*** |

HF, heart failure; LD, loop diuretic

*p<0·05 **p<0·005 ***p<0·0005.

^Δ^ Hazard ratios (95% CI) are derived using the method of Fine and Gray, adjusting for death as a competing risk.

^a^ Model 1 is the unadjusted analysis

^b^ Model 2 adjusts for age, gender, systolic blood pressure, BMI, smoking status, and comorbidities at

AF diagnosis: COPD, diabetes, hypertension, previous MI, previous stroke/TIA.

^c^ Model 3 adjusts for variables in Model 2 + medication use at AF diagnosis (ACEI/ARB, beta-blocker, MRA, digoxin, anticoagulant, antiplatelet, antiarrhythmic drug, non-dihydropyridine CCB, and statin use).

**Supplemental Table 6. Risk of adverse outcomes after AF diagnosis by baseline heart failure status***. Including only patients with non-valvular AF (n=109,844).*

| Outcome | Model 1^a^ | Model 2^b^ | Model 3^c^ |
| --- | --- | --- | --- |
|  | **HR (95% CI)** | **HR (95% CI)** | **HR (95% CI)** |
| All-cause mortality |  |  |  |
| No HF or LD use | Referent | Referent | Referent |
| Isolated LD use | 2·14 (2·07-2·20)*** | 1·45 (1·40-1·51)*** | 1·41 (1·37-1·47)*** |
| Diagnosed HF | 1·56 (1·47-1·65)*** | 1·35 (1·27-1·45)*** | 1·32 (1·24-1·42)*** |
| Diagnosed HF + LD use | 2·80 (2·72-2·89)*** | 1·90 (1·83-1·97)*** | 1·76 (1·68-1·83)*** |
| Unplanned HF hospitalisation ^Δ^ |  |  |  |
| No HF or LD use | Referent – was 3 | Referent | Referent |
| Isolated LD use | 1·91 (1·72-2·12)*** | 1·61 (1·42-1·82)*** | 1·59 (1·40-1·81)*** |
| Diagnosed HF | 3·53 (3·05-4·09)*** | 3·22 (2·73-3·81)*** | 3·21 (2·71-3·81)*** |
| Diagnosed HF + LD use | 3·70 (3·35-4·08)*** | 2·89 (2·55-3·28)*** | 2·78 (2·41-3·20)*** |
| Ischaemic stroke ^Δ^ | | | |
| No HF or LD use | Referent | Referent | Referent |
| Isolated LD use | 0·98 (0·93-1·03) | 0·85 (0·80-0·90)*** | 0·87 (0·82-0·92)*** |
| Diagnosed HF | 0·99 (0·89-1·09) | 1·02 (0·91-1·14) | 1·04 (0·93-1·17) |
| Diagnosed HF + LD use | 0·80 (0·75-0·86)*** | 0·72 (0·66-0·78)*** | 0·76 (0·70-0·83)*** |

HF, heart failure; LD, loop diuretic

*p<0·05 **p<0·005 ***p<0·0005.

^Δ^ Hazard ratios (95% CI) are derived using the method of Fine and Gray, adjusting for death as a competing risk.

^a^ Model 1 is the unadjusted analysis

^b^ Model 2 adjusts for age, gender, systolic blood pressure, BMI, smoking status, and comorbidities at

AF diagnosis: COPD, diabetes, hypertension, previous MI, previous stroke/TIA.

^c^ Model 3 adjusts for variables in Model 2 + medication use at AF diagnosis (ACEI/ARB, beta-blocker, MRA, digoxin, anticoagulant, antiplatelet, antiarrhythmic drug, non-dihydropyridine CCB, and statin use).

**Supplemental Table 7.** Adverse outcomes after AF diagnosis by baseline heart failure status. *Including outcomes occurring within 100-days of AF diagnosis.*

| Outcome | Total population  n=124,256 | Status at time of AF diagnosis | | | |
| --- | --- | --- | --- | --- | --- |
|  |  | **No heart failure diagnosis**  **n=102,255** | | **Diagnosed heart failure**  **n=22,001** | |
|  |  | **No loop diuretic**  **n=79,930** | **Isolated loop diuretic use**  **n=22,235** | **No loop diuretic**  **n=6,082** | **Loop diuretic**  **n=15,919** |
| All-cause mortality |  |  |  |  |  |
| No. events, n (%) | 36,519 (29·3) | 18,204 (22·8) | 8,648 (38·7) | 2,085 (34·3) | 7,582 (47·6) |
| No. events <100-days of AF diagnosis, n (%) | 4,216 (3·4) | 2,118 (2·6) | 797 (3·6) | 469 (7·7) | 832 (5·2) |
| Unplanned HF hospitalisation |  |  |  |  |  |
| No. events, n (%) | 4,018 (3·2) | 1,246 (1·6) | 658 (3·0) | 553 (9·1) | 1,561 (9·8) |
| No. events <100-days of AF diagnosis, n (%) | 935 (0·8) | 8 (0·01) | 5 (0·02) | 272 (4·5) | 650 (4·1) |
| Ischaemic stroke/TIA |  |  |  |  |  |
| No. events, n (%) | 16,026 (12·9) | 10,686 (13·4) | 2,845 (12·7) | 791 (13·0) | 1,704 (10·7) |
| No. events <100-days of AF diagnosis, n (%) | 4,403 (3·5) | 3,000 (3·8) | 710 (3·2) | 259 (4·3) | 434 (2·7) |

HF, heart failure; TIA, transient ischaemic attack

**Supplemental Table 8. Risk of adverse outcomes after AF diagnosis by baseline heart failure status***. No censor period after AF diagnosis (i.e. including outcomes occurring within 100 days of AF diagnosis)*

| Outcome | Model 1^a^ | Model 2^b^ | Model 3^c^ |
| --- | --- | --- | --- |
|  | **HR (95% CI)** | **HR (95% CI)** | **HR (95% CI)** |
| All-cause mortality |  |  |  |
| No HF or LD use | Referent | Referent | Referent |
| Isolated LD use | 2·00 (1·95-2·05) *** | 1·40 (1·36-1·45) *** | 1·39 (1·34-1·43) *** |
| Diagnosed HF | 1·74 (1·67-1·82) *** | 1·47 (1·40-1·56) *** | 1·46 (1·38-1·54) *** |
| Diagnosed HF + LD use | 2·67 (2·60-2·75) *** | 1·86 (1·80-1·93) *** | 1·74 (1·68-1·81) *** |
| Unplanned HF hospitalisation ^Δ^ |  |  |  |
| No HF or LD use | Referent | Referent | Referent |
| Isolated LD use | 1·88 (1·72-2·07)*** | 1·70 (1·52-1·90)*** | 1·61 (1·44-1·81)*** |
| Diagnosed HF | 6·28 (5·68-6·95)*** | 5·53 (4·91-6·22)*** | 5·30 (4·69-5·98)*** |
| Diagnosed HF + LD use | 6·53 (6·06-7·03)*** | 5·46 (4·97-6·00)*** | 4·85 (4·36-5·40)*** |
| Ischaemic stroke ^Δ^ |  |  |  |
| No HF or LD use | Referent | Referent | Referent |
| Isolated LD use | 0·94 (0·90-0·98)** | 0·82 (0·78-0·87)*** | 0·85 (0·80-0·89)*** |
| Diagnosed HF | 0·99 (0·92-1·06) | 1·03 (0·95-1·13) | 1·06 (0·97-1·16) |
| Diagnosed HF + LD use | 0·77 (0·73-0·81)*** | 0·72 (0·67-0·76)*** | 0·77 (0·72-0·83)*** |

HF, heart failure; LD, loop diuretic

*p<0·05 **p<0·005 ***p<0·0005.

^Δ^ Hazard ratios (95% CI) are derived using the method of Fine and Gray, adjusting for death as a competing risk.

^a^ Model 1 is the unadjusted analysis

^b^ Model 2 adjusts for age, gender, systolic blood pressure, BMI, smoking status, and comorbidities at

AF diagnosis: COPD, diabetes, hypertension, previous MI, previous stroke/TIA.

^c^ Model 3 adjusts for variables in Model 2 + medication use at AF diagnosis (ACEI/ARB, beta-blocker, MRA, digoxin, anticoagulant, antiplatelet, antiarrhythmic drug, non-dihydropyridine CCB, and statin use).

**Supplemental Table 9. Risk of adverse outcomes after AF diagnosis by baseline heart failure status***. Increasing the censor period to 1 year.*

| Outcome | Model 1^a^ | Model 2^b^ | Model 3^c^ |
| --- | --- | --- | --- |
|  | **HR (95% CI)** | **HR (95% CI)** | **HR (95% CI)** |
| All-cause mortality |  |  |  |
| No HF or LD use | Referent | Referent | Referent |
| Isolated LD use | 2·12 (2·06-2·19)*** | 1·47 (1·41-1·52)*** | 1·43 (1·38-1·49)*** |
| Diagnosed HF | 1·48 (1·39-1·57)*** | 1·32 (1·23-1·41)*** | 1·29 (1·21-1·38)*** |
| Diagnosed HF + LD use | 2·75 (2·66-2·84)*** | 1·91 (1·84-1·99)*** | 1·77 (1·70-1·85)*** |
| Unplanned HF hospitalisation ^Δ^ |  |  |  |
| No HF or LD use | Referent | Referent | Referent |
| Isolated LD use | 1·92 (1·73-2·14)*** | 1·58 (1·39-1·80)*** | 1·57 (1·37-1·79)*** |
| Diagnosed HF | 3·06 (2·62-3·57)*** | 2·68 (2·25-3·19)*** | 2·67 (2·23-3·20)*** |
| Diagnosed HF + LD use | 3·63 (3·28-3·01)*** | 2·83 (2·50-3·21)*** | 2·77 (2·40-3·19)*** |
| Ischaemic stroke ^Δ^ | | | |
| No HF or LD use | Referent | Referent | Referent |
| Isolated LD use | 1·00 (0·94-1·06) | 0·86 (0·80-0·92)*** | 0·88 (0·82-0·95)*** |
| Diagnosed HF | 1·01 (0·91-1·12) | 1·00 (0·88-1·12) | 1·03 (0·92-1·16) |
| Diagnosed HF + LD use | 0·82 (0·76-0·88)*** | 0·72 (0·67-0·79)*** | 0·78 (0·71-0·86)*** |

HF, heart failure; LD, loop diuretic

*p<0·05 **p<0·005 ***p<0·0005.

^Δ^ Hazard ratios (95% CI) are derived using the method of Fine and Gray, adjusting for death as a competing risk.

^a^ Model 1 is the unadjusted analysis

^b^ Model 2 adjusts for age, gender, systolic blood pressure, BMI, smoking status, and comorbidities at

AF diagnosis: COPD, diabetes, hypertension, previous MI, previous stroke/TIA.

^c^ Model 3 adjusts for variables in Model 2 + medication use at AF diagnosis (ACEI/ARB, beta-blocker, MRA, digoxin, anticoagulant, antiplatelet, antiarrhythmic drug, non-dihydropyridine CCB, and statin use).

**Supplemental Table 10. Risk of adverse outcomes after AF diagnosis by baseline heart failure status***. Excluding patients initiated on loop-diuretic therapy in the last 12 months of life.*

| Outcome | Model 1^a^ | Model 2^b^ | Model 3^c^ |
| --- | --- | --- | --- |
|  | **HR (95% CI)** | **HR (95% CI)** | **HR (95% CI)** |
| All-cause mortality |  |  |  |
| No HF | Referent | Referent | Referent |
| Possible HF | 1.94 (1.89-2.00)*** | 1.39 (1.34-1.43)*** | 1.35 (1.31-1.40)*** |
| Diagnosed HF – loop diuretic | 1.55 (1.47-1.63)*** | 1.38 (1.30-1.46)*** | 1.35 (1.27-1.43)*** |
| Diagnosed HF + loop diuretic | 2.60 (2.52-2.68)*** | 1.86 (1.79-1.92)*** | 1.70 (1.64-1.77)*** |
| Unplanned HF hospitalisation ^Δ^ |  |  |  |
| No HF | Referent | Referent | Referent |
| Possible HF | 1.91 (1.74-2.10)*** | 1.63 (1.45-1.83)*** | 1.60 (1.42-1.80)*** |
| Diagnosed HF – loop diuretic | 3.39 (2.97-3.86)*** | 3.04 (2.62-3.53)*** | 3.00 (2.58-3.50)*** |
| Diagnosed HF + loop diuretic | 4.01 (3.68-4.37)*** | 3.22 (2.89-3.59)*** | 3.03 (2.68-3.43)*** |
| Ischaemic stroke ^Δ^ | | | |
| No HF | Referent | Referent | Referent |
| Possible HF | 1.00 (0.96-1.05) | 0.87 (0.82-0.92)*** | 0.89 (0.84-0.95)*** |
| Diagnosed HF – loop diuretic | 0.98 (0.90-1.07) | 1.00 (0.90-1.10) | 1.02 (0.92-1.13) |
| Diagnosed HF + loop diuretic | 0.82 (0.78-0.88)*** | 0.74 (0.69-0.79)*** | 0.79 (0.74-0.86)*** |

HF, heart failure; LD, loop diuretic

*p<0·05 **p<0·005 ***p<0·0005.

^Δ^Hazard ratios (95% CI) are derived using the method of Fine and Gray, adjusting for death as a competing risk.

^a^ Model 1 is the unadjusted analysis

^b^ Model 2 adjusts for age, gender, systolic blood pressure, BMI, smoking status, and comorbidities at

AF diagnosis: COPD, diabetes, hypertension, previous MI, previous stroke/TIA.

^c^ Model 3 adjusts for variables in Model 2 + medication use at AF diagnosis (ACEI/ARB, beta-blocker, MRA, digoxin, anticoagulant, antiplatelet, antiarrhythmic drug, non-dihydropyridine CCB, and statin use).

**Supplemental Table 11. Risk of adverse outcomes after AF diagnosis by baseline heart failure status***. Restricted to patients with no BMI data available.*

| Outcome | Model 1^a^ | Model 2^b^ | Model 3^c^ |
| --- | --- | --- | --- |
|  | **HR (95% CI)** | **HR (95% CI)** | **HR (95% CI)** |
| All-cause mortality |  |  |  |
| No HF | Referent | Referent | Referent |
| Possible HF | 2.52 (2.39-2.65)*** | 1.42 (1.34-1.50)*** | 1.39 (1.31-1.47)*** |
| Diagnosed HF – loop diuretic | 1.69 (1.53-1.88)*** | 1.38 (1.24-1.53)*** | 1.33 (1.19-1.47)*** |
| Diagnosed HF + loop diuretic | 3.11 (2.94-3.30)*** | 1.79 (1.68-1.90)*** | 1.64 (1.53-1.76)*** |
| Unplanned HF hospitalisation ^Δ^ |  |  |  |
| No HF | Referent | Referent | Referent |
| Possible HF | 2.08 (1.69-2.56)*** | 1.66 (1.33-2.08)*** | 1.59 (1.27-2.00)*** |
| Diagnosed HF – loop diuretic | 3.39 (2.51-4.58)*** | 3.02 (2.22-4.13)*** | 2.89 (2.12-3.95)*** |
| Diagnosed HF + loop diuretic | 4.40 (3.63-5.33)*** | 3.38 (2.73-4.19)*** | 3.06 (2.40-3.94)*** |
| Ischaemic stroke ^Δ^ | | | |
| No HF | Referent | Referent | Referent |
| Possible HF | 0.98 (0.87-1.09) | 0.75 (0.66-0.84)*** | 0.74 (0.66-0.84)*** |
| Diagnosed HF – loop diuretic | 1.00 (0.81-1.22) | 0.88 (0.71-1.09) | 0.88 (0.71-1.09) |
| Diagnosed HF + loop diuretic | 0.89 (0.78-1.03) | 0.68 (0.59-0.79)*** | 0.69 (0.59-0.80)*** |

HF, heart failure; LD, loop diuretic

*p<0·05 **p<0·005 ***p<0·0005.

^Δ^Hazard ratios (95% CI) are derived using the method of Fine and Gray, adjusting for death as a competing risk.

^a^ Model 1 is the unadjusted analysis

^b^ Model 2 adjusts for age, gender, systolic blood pressure, smoking status, and comorbidities at AF diagnosis: COPD, diabetes, hypertension, previous MI, previous stroke/TIA.

^c^ Model 3 adjusts for variables in Model 2 + medication use at AF diagnosis (ACEI/ARB, beta-blocker, MRA, digoxin, anticoagulant, antiplatelet, antiarrhythmic drug, non-dihydropyridine CCB, and statin use).

**Supplemental Table 12. Risk of adverse outcomes after AF diagnosis by baseline heart failure status***. Re-classifying patients with stage 3 and 4 chronic kidney disease (n=1,257), in addition to stage 5 as per the main analysis, as having a possible non-cardiac indication for loop-diuretic therapy (in addition to those with stage 5 and renal replacement therapy).*

| Outcome | Model 1^a^ | Model 2^b^ | Model 3^c^ |
| --- | --- | --- | --- |
|  | **HR (95% CI)** | **HR (95% CI)** | **HR (95% CI)** |
| All-cause mortality |  |  |  |
| No HF | Referent | Referent | Referent |
| Possible HF | 1.89 (1.84-1.94) *** | 1.37 (1.32-1.41) *** | 1.34 (1.30-1.38) *** |
| Diagnosed HF – loop diuretic | 2.24 (2.16-2.32) *** | 1.82 (1.74-1.90) *** | 1.73 (1.65-1.81) *** |
| Diagnosed HF + loop diuretic | 2.40 (2.33-2.47) *** | 1.74 (1.68-1.80) *** | 1.60 (1.54-1.67) *** |
| Unplanned HF hospitalisation ^Δ^ |  |  |  |
| No HF | Referent | Referent | Referent |
| Possible HF | 2.09 (1.90-2.30)*** | 1.66 (1.48-1.85)*** | 1.57 (1.40-1.77)*** |
| Diagnosed HF – loop diuretic | 8.06 (7.39-8.80)*** | 5.61 (5.05-6.24)*** | 5.23 (4.68-5.84)*** |
| Diagnosed HF + loop diuretic | 7.47 (6.92-8.07)*** | 5.24 (4.76-5.78)*** | 4.66 (4.18-5.20)*** |
| Ischaemic stroke ^Δ^ | | | |
| No HF | Referent | Referent | Referent |
| Possible HF | 0.93 (0.89-0.97)** | 0.83 (0.79-0.87)*** | 0.85 (0.81-0.90)*** |
| Diagnosed HF – loop diuretic | 0.95 (0.89-1.01) | 0.92 (0.85-0.99)* | 0.96 (0.88-1.04) |
| Diagnosed HF + loop diuretic | 0.75 (0.71-0.80)*** | 0.73 (0.69-0.78)*** | 0.79 (0.74-0.85)*** |

HF, heart failure; LD, loop diuretic

*p<0·05 **p<0·005 ***p<0·0005.

^Δ^Hazard ratios (95% CI) are derived using the method of Fine and Gray, adjusting for death as a competing risk.

^a^ Model 1 is the unadjusted analysis

^b^ Model 2 adjusts for age, gender, systolic blood pressure, BMI, smoking status, and comorbidities at

AF diagnosis: COPD, diabetes, hypertension, previous MI, previous stroke/TIA.

^c^ Model 3 adjusts for variables in Model 2 + medication use at AF diagnosis (ACEI/ARB, beta-blocker, MRA, digoxin, anticoagulant, antiplatelet, antiarrhythmic drug, non-dihydropyridine CCB, and statin use).

**Supplemental Table 13. Risk of adverse outcomes after AF diagnosis by heart failure status at any time during follow-up***. Exposure categories defined using a time-dependent approach (i.e., isolated loop-diuretic use or HF diagnosis occurring at any time during follow-up).*

| Outcome | Model 1^a^ | Model 2^b^ | Model 3^c^ |
| --- | --- | --- | --- |
|  | **HR (95% CI)** | **HR (95% CI)** | **HR (95% CI)** |
| All-cause mortality |  |  |  |
| No HF | Referent | Referent | Referent |
| Possible HF | 2.12 (2.04-2.19)*** | 1.42 (1.36-1.48)*** | 1.37 (1.31-1.44)*** |
| Diagnosed HF – loop diuretic | 2.03 (1.91-2.16)*** | 1.45 (1.34-1.57)*** | 1.41 (1.30-1.53)*** |
| Diagnosed HF + loop diuretic | 2.86 (2.75-2.97)*** | 1.97 (1.88-2.06)*** | 1.80 (1.71-1.89)*** |
| Unplanned HF hospitalisation ^Δ^ |  |  |  |
| No HF | Referent | Referent | Referent |
| Possible HF | 2.32 (2.04-2.64)*** | 1.77 (1.52-2.05)*** | 1.72 (1.48-2.00)*** |
| Diagnosed HF – loop diuretic | 4.41 (3.72-5.22)*** | 3.88 (3.21-4.69)*** | 3.79 (3.13-4.59)*** |
| Diagnosed HF + loop diuretic | 6.13 (5.48-6.86)*** | 4.34 (3.80-4.95)*** | 3.98 (3.45-4.60)*** |
| Ischaemic stroke ^Δ^ | | | |
| No HF | Referent | Referent | Referent |
| Possible HF | 1.14 (1.07-1.21)*** | 0.92 (0.86-0.99)* | 0.94 (0.88-1.01) |
| Diagnosed HF – loop diuretic | 1.07 (0.96-1.19) | 1.07 (0.95-1.20) | 1.09 (0.96-1.23) |
| Diagnosed HF + loop diuretic | 1.01 (0.94-1.08) | 0.83 (0.76-0.90)*** | 0.87 (0.80-0.95)** |

HF, heart failure; LD, loop diuretic

*p<0·05 **p<0·005 ***p<0·0005.

^Δ^Hazard ratios (95% CI) are derived using the method of Fine and Gray, adjusting for death as a competing risk.

^a^ Model 1 is the unadjusted analysis

^b^ Model 2 adjusts for age, gender, systolic blood pressure, BMI, smoking status, and comorbidities at

AF diagnosis: COPD, diabetes, hypertension, previous MI, previous stroke/TIA.

^c^ Model 3 adjusts for variables in Model 2 + medication use at AF diagnosis (ACEI/ARB, beta-blocker, MRA, digoxin, anticoagulant, antiplatelet, antiarrhythmic drug, non-dihydropyridine CCB, and statin use).

**Supplemental figure legends**

**Supplemental figure 1.** Prevalence of diagnosed heart failure and isolated loop-diuretic use at AF diagnosis by study year. Includes heart failure diagnosed prior to [>3 months] and concomitant with [±3months] AF diagnosis.

**Supplemental figure 2.** Prevalence of diagnosed heart failure and isolated loop-diuretic use at AF diagnosis by gender. A. Women, B. Men. Includes heart failure diagnosed prior to [>3 months] and concomitant with [±3months] AF diagnosis.

**Supplemental figure 3.** Cumulative incidence of (A) diagnosed heart failure and (B) diagnosed heart failure and isolated loop-diuretic use combined, and the competing risk of death before heart failure, after AF diagnosis. Study cohort comprises patients without heart failure or loop-diuretic use at AF diagnosis.

**Supplemental figure 4.** Survival after AF diagnosis, stratified by gender and baseline heart failure status. **A.** Diagnosed heart failure versus no heart failure. **B.** Diagnosed heart failure and isolated loop-diuretic use (combined) versus no heart failure. **excluding a 100-day censor period*

**Supplemental figure 1.**

**Supplemental figure 2.**

**Supplemental figure 3.**

**Supplemental figure 4**

**
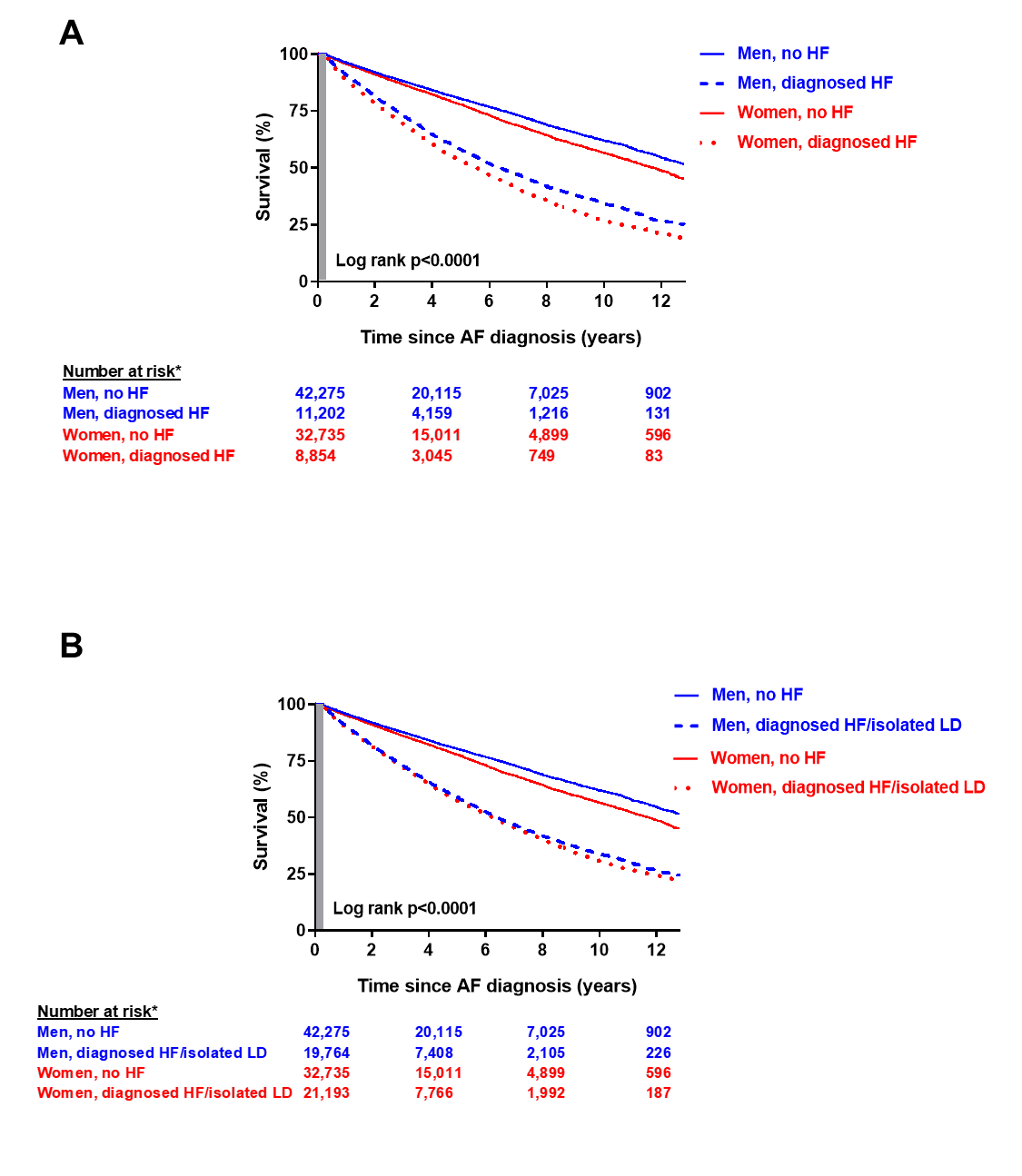
**
